# Identification of Altered Primary Immunodeficiency-Associated Genes and Their Implications in Pediatric Cancers

**DOI:** 10.3390/cancers14235942

**Published:** 2022-11-30

**Authors:** Shaelene Standing, Son Tran, Luis Murguia-Favela, Olga Kovalchuk, Pinaki Bose, Aru Narendran

**Affiliations:** 1 Section of Pediatric Oncology and Blood and Marrow Transplantation, Division of Pediatrics, Alberta Children’s Hospital and University of Calgary, Calgary, AB T3B 6A8, Canada; 2 Section of Pediatric Hematology and Immunology, Division of Pediatrics, Alberta Children’s Hospital and University of Calgary, Calgary, AB T3B 6A8, Canada; 3 Department of Biological Sciences, University of Lethbridge, Lethbridge, AB T1K 3M4, Canada; 4 Departments of Oncology, Biochemistry and Molecular Biology, University of Calgary, Calgary, AB T2N 1N4, Canada

**Keywords:** pediatric cancer, immunodeficiency, differential expression, survival, oncogenesis

## Abstract

**Simple Summary:**

In children, cancer remains the most common cause of disease-related mortality and is responsible for more deaths from infancy through adolescence than any other disease. Malignancies are observed more frequently in individuals with primary immunodeficiencies (PID), and cancer is one of the most common causes of death in patients with PIDs. However, the molecular mechanisms that link the immune function to malignancy development remain poorly understood. The primary aim of this project was to identify and highlight the molecular mechanisms by which PID-related genes may lead to the development of pediatric cancers and was completed using a novel bioinformatics framework. This study highlighted multiple PID-related genes for further investigation regarding their implications in PIDs and pediatric cancer mechanisms which may lead to the identification of new therapeutic targets.

**Abstract:**

Background: Cancer is the leading cause of disease-related mortality in children and malignancies are more frequently observed in individuals with primary immunodeficiencies (PIDs). This study aimed to identify and highlight the molecular mechanisms, such as oncogenesis and immune evasion, by which PID-related genes may lead to the development of pediatric cancers. Method: We implemented a novel bioinformatics framework using patient data from the TARGET database and performed a comparative transcriptome analysis of PID-related genes in pediatric cancers between normal and cancer tissues, gene ontology enrichment, and protein–protein interaction analyses, and determined the prognostic impacts of commonly mutated and differentially expressed PID-related genes. Results: From the Fulgent Genetics Comprehensive Primary Immunodeficiency panel of 472 PID-related genes, 89 genes were significantly differentially expressed between normal and cancer tissues, and 20 genes were mutated in two or more patients. Enrichment analysis highlighted many immune system processes as well as additional pathways in the mutated PID-related genes related to oncogenesis. Survival outcomes for patients with altered PID-related genes were significantly different for 75 of the 89 DEGs, often resulting in a poorer prognosis. Conclusions: Overall, multiple PID-related genes demonstrated the connection between PIDs and cancer development and should be studied further, with hopes of identifying new therapeutic targets.

## 1. Introduction

Cancer remains the most common cause of disease-related mortality in children, and it is responsible for more deaths from infancy through adolescence than any other disease [1]. Malignancies in children are more aggressive and invasive than tumors in adults [1]. Cancers also occur more often in children at different sites and are more likely to be hemopoietic or embryonic in origin, with leukemia being the most commonly diagnosed cancer in children [1]. Malignancies are observed more frequently in individuals with primary immunodeficiencies (PIDs; also known as inborn errors of immunity (IEI)), and cancer is one of the most common causes of death in patients with PIDs [2,3]. In addition, several well-known PID-related genes are recognized as cancer-predisposing genes, including those found in immune dysregulation (e.g., ITK, KRAS), combined immunodeficiencies (e.g., JAK3, RECQL4) and phagocytic disorders (e.g., CDKN2A, CSF3R) [2]. However, the molecular mechanisms that link immune function to malignancy development remain poorly understood. The concept of immune surveillance postulates that the immune system plays a critical role in oncogenesis through immune cells’ detection and elimination of tumor cells in the body [3]. Thus, maintaining the integrity of the immune system is critical in the surveillance of abnormal cell growth and survival. In fact, tumor surveillance has been noted as a hallmark of cancer [3,4,5]. The theory of immune surveillance predicts that PIDs are an important contributor to cancer development and outcomes due to the alteration or depletion of immune cell function [3]. Furthermore, the molecular genetic defects that are thought to underlie PID are also found in cancer predisposition [3].

**Figure 1:**
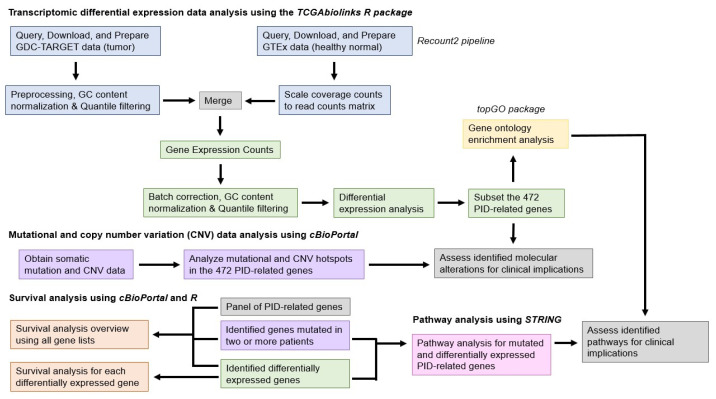
Workflow illustrating steps used in this study to identify the altered molecular characteristics of PID-related genes in pediatric cancer tumor tissues.

Recent studies have identified over 485 PID-related genes [6]. Pathways and mechanisms by which genetic alterations in PID-related genes may lead to cancers in children have also been identified. This research is based on previous preliminary studies from our laboratory that have identified a group of immunodeficiency-associated gene mutations in pediatric tumors, plus a related study investigating PID-related genes in adult malignancies [7,8]. The primary aim of this project was to identify and highlight the molecular mechanisms by which PID-related genes may lead to the development of pediatric cancers. The project employed a novel bioinformatics framework (Figure 1). Our first objective was to investigate the differential expression (between normal and cancer tissues) of PID-related genes in pediatric cancers through comparative transcriptome analysis, in addition to assessing the mutational frequency of PID-related genes in pediatric cancers. Our second objective was to analyze common pathways between mutated and differentially expressed genes using gene ontology (GO) enrichment and protein–protein interaction (PPI) analyses to better illustrate the molecular mechanisms involved in immune function and pediatric cancer development. Finally, we determined the prognostic impacts of commonly mutated and differentially expressed PID-related genes through a comprehensive survival analysis. Given the intrinsic factors that underlie cancer predisposition in PIDs, we hypothesize that molecular abnormalities in PID-related genes exist in pediatric cancers, thus impacting immune function and patient outcomes.

## 2. Materials and Methods

### 2.1. Data Collection and Processing

The TCGAbiolinks R package was used to download and process publicly available genomic data from the Therapeutically Applicable Research to Generate Effective Treatments (TARGET) database within the Genomic Data Commons (GDC) Data Portal for six different pediatric tumors [9,10]—acute myeloid leukemia (AML; *n* = 2245), acute lymphocytic leukemia (ALL; *n* = 463), neuroblastoma (NBL; *n* = 153), Wilms’ tumor (WT; *n* = 124), rhabdoid tumor (RT; *n* = 63), clear cell sarcoma of the kidney (CCSK; *n* = 13). Each available tumor sample of each cancer type was analyzed separately. Two adult healthy tissue samples were used as control (normal tissue comparison) from the Genotype-Tissue Expression (GTEx) Project. The Recount2 pipeline was used to compare expression among tumor samples [9,11]. GTEx blood normal tissue samples were used as control for ALL and AML whereas GTEx kidney normal tissue samples were used as control for CCSK, NBL, RT, and WT. The integration of the GTEx adult normal tissue samples with the TARGET tissue samples have been justified due to samples being processed in the same manner as highlighted by previously published work [12,13]. TARGET tumor samples were preprocessed and normalized for GC content and quantile filtered using the TCGAanalyze_Preprocessing, TCGAanalyze_Normalization, and TCGAanalyze_Filtering functions from the TCGAbiolinks package [9]. Batch correction was performed on the TARGET tumor and the GTEx healthy tissue samples using the TCGAbiolinks TCGAbatch_Correction function, and then was normalized for GC content and filtered by quantile [9].

### 2.2. Differential Gene Expression Analysis of Unified TARGET-GTEx Datasets

Differential gene expression analysis (DEA) was performed using the TCGAanalyze_DEA function in the *TCGAbiolinks* package, using both the *limma-voom* and *edgeR* pipelines which have been used previously to analyze genes that were differentially expressed in tumor samples in comparison to healthy tissue samples [9,14,15]. Log fold change (logFC) > |±2.0| and false discovery rate adjusted *p*-value (FDR adj. *p.*) < 10 × 10^−16^ were defined as cut-off values to retain significant differentially expressed genes (DEGs) for the analysis. Differentially expressed genes (DEGs) were then compared to a subset of 472 PID-related genes curated from the Fulgent Genetics Comprehensive Primary Immunodeficiency (NGS) panel [16]. The Fulgent Genetics NGS panel contained the greatest number of PID-related genes in comparison to other publicly available gene panels for PID-related genes [16]. The subset of DEGs that were noted to be PID-related genes was visualized using the *EnhancedVolcano* R package [14]. DEA results from all the pediatric tumor samples for each pipeline were summarized and visualized. For this study, trends in AML and ALL cancer types included the results from the TB (primary blood-derived cancer peripheral blood tissue) and TBM (primary blood-derived cancer bone marrow tissue) tissues in the TARGET database. For all other cancers, the TP (primary tumor tissue) sample type was available and used. Multiple ALL phases were also available, however, only ALL-P2 produced significant results with the *limma-voom* pipeline and only ALL-P1 produced significant results with the *edgeR* pipeline, and these were grouped when looking at overall DEA trends. Genes that were commonly significantly altered were further investigated to assess their biological and clinical significance in pediatric cancers.

### 2.3. Mutational and Copy Number Variation Analysis

Mutational analysis was performed in the five available TARGET pediatric cancer cohorts from the cBioPortal [17]. The cancers included in the mutational analysis were ALL, AML, NBL, RT, and WT, and the genes queried included the panel of 472 PID-related genes. Genes that were mutated in at least two of the 2087 patient profiles were analyzed further regarding the types of mutations and visualized using the *ggplot2* package in R as well as investigated regarding their clinical significance. It is important to note that none of the genes were mutated in more than 1% of total patients which may be partially due to the limited data available in pediatric cancer cohorts, and therefore, our analysis is limited. Protein changes in all cancer types were extracted from cBioPortal [17]. Copy number variant analysis was performed by obtaining the copy number variation data from the four TARGET pediatric cancer cohort projects with this data available including ALL, AML, NBL, and WT on cBioPortal [17].

### 2.4. Functional Enrichment Analysis

Genes that were significantly altered in the DEA were analyzed further through a Gene Ontology (GO) enrichment analysis using the *topGO* package in R to assess the altered molecular pathways for the DEGs [18]. GO enrichment analysis regarding biological process (BP) terms used Kolmogorov–Smirnov (KS) statistical testing with KS < 0.05 and enrichment ≥ 2. GO enrichment analysis results were visualized using the *ggplot2* package in R.

STRING (v11) was used for pathway analysis for the 89 significantly DEGs and 20 genes mutated in two or more patients to determine protein interactions important in the cancer profiles [19]. The enrichment *p*-values and BP GO terms were assessed to identify and validate pathways found in the earlier GO analysis.

### 2.5. Survival Analysis

Using cBioPortal, survival analysis was performed by obtaining the survival data from the following available pediatric tissue samples: ALL, AML, NBL, RT, and WT [17]. The altered group for this analysis is defined as harboring somatic mutations from whole genome or whole exome sequencing. Kaplan–Meier survival curves and associated log-rank test *p*-values were generated from the analysis. Survival analysis was performed using two different gene queries: the panel of 472 PID-related genes, and the 20 PID-related genes mutated in two or more patients. Significant survival difference was determined using a *p*-value of <0.05. Following this, survival analysis using overall survival as an outcome was completed for each DEG for each pediatric cancer type with TARGET data publicly available. Using an R script, Kaplan–Meier survival curves were generated to assess the clinical impact of significant PID-related DEGs in pediatric cancers, and those with significant Cox regression results were identified (*p* < 0.05). The cut-off points for high- and low-expressing groups were determined by tertial splitting (retaining the samples with expression levels above the 67th percentile or below the 33rd percentile, respectively).

## 3. Results

### 3.1. Differential Expression of PID-Related Genes in Pediatric Cancer Datasets

**Figure 2:**
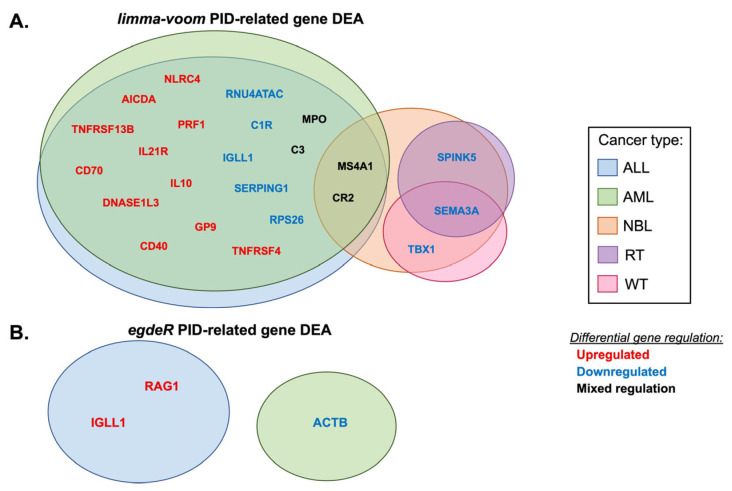
Venn diagram summary of DEA results of PID-related genes that are significantly differentially expressed in pediatric cancer types including AML, ALL, WT, RT, and NBL. Differentially expressed genes in each cancer type are listed (red: upregulated, blue: downregulated, or black: if the gene had mixed regulation in different cancer types. The analyses have been performed using the TCGAbiolinks R package and the (**A**) limma-voom pipeline, or (**B**) edgeR pipeline.

*Limma-voom* (Figure S1) and edgeR (Figure S2) pipelines were individually analyzed for each pediatric cancer type, and the significantly altered genes were extracted. Overall, 89 of the PID-related genes were differentially expressed in at least one pipeline. Of the 472 PID-related genes, 88 were differentially expressed in at least one pediatric cancer type when using the *limma-voom* pipeline (Figure S3). Furthermore, 23 PID-related genes were differentially expressed in at least two different pediatric cancer types when using the *limma-voom* pipeline (Figure 2A). The three most upregulated genes were *AICDA*, *TNFRSF13B*, and *CR2* (Table S1). The three most downregulated genes were *SEMA3A*, *GATA2*, and *CFTR* (Table S1). The three genes that were most commonly altered were *CR2*, *MS4A1*, and *SEMA3A*. (Figure 2A). *CR2* and *MS4A1* were both differentially expressed in NBL, AML, and ALL, whereas *SEMA3A* was differentially expressed in RT, NBL, and WT. *SEMA3A* was downregulated in all the cancers in which it was differentially expressed, while the regulation of *CR2* and *MS4A1* varied according to cancer type (Figure 2A). *MS4A1* was upregulated in AML and ALL (TBM) samples and downregulated in NBL and ALL (TB) samples (Table S1). *CR2* was upregulated in AML and downregulated in both NBL and ALL (Table S1).

Using the edgeR pipeline, only three PID genes were differentially expressed: *ACTB, IGLL1,* and *RAG1* (Figure 2B). *IGLL1* and *RAG1* were upregulated in ALL, whereas *ACTB* was downregulated in AML (Table S1). *RAG1* and *IGLL1* were the only two genes that were differentially expressed when using both the *limma-voom* and *egdeR* pipelines, indicating that additional genes may be strong candidates for further exploration due to their consistent significant differential expression.

### 3.2. PID-Related DEGs Are Associated with Various Immunological and Oncologic Pathways

GO enrichment analysis of DEGs overall (Table S2) and the subset of the PID-related DEGs (Table S3) identified multiple molecular mechanisms that were significantly altered. The identified individual GO terms were grouped into overarching themes. For all the genes that were differentially expressed, metabolic molecular mechanisms were most commonly altered in pediatric cancers, followed by immune system function, developmental processes, vesicle and ion transport, and transcription regulation. For the subset of differentially expressed PID-related genes, immune system functioning was the only common theme identified, as would be expected, owing to the impact of these genes on PIDs. Innate immunity was significantly impacted in all the pediatric cancers studied. Adaptive immune responses and development were identified in ALL and AML, respectively. The only cancers that had significant PID-related gene enrichment analysis results were AML and ALL (Table S3).

STRING was used to assess the gene interaction networks of the 89 and 20 genes highlighted by the DEA and mutational analysis, respectively. A significant enrichment *p*-value was identified for the gene lists of both the DEGs and genes mutated in two or more patients, demonstrating that the proteins encoded by these genes have significant co-interactions (Figures S6 and S7 and Tables S7 and S8). Network analyses of the DE genes mutated in two or more patients produced similar results using STRING when assessing the identified GO enrichment of biological processes (Tables S7 and S8). Interestingly, these results identified GO terms similar to those identified in the earlier GO analysis of the DEGs when observing each cancer type individually (Table S3). Most of the terms were related to the immune system, particularly immune system processes (GO:0002376), responses to stimuli or other organisms (GO:0051707/GO:0050896), and immune system responses (GO:0006955; Tables S7 and S8). For the mutated genes, pathways related to proliferation, differentiation, signaling, and cell motility were also commonly observed, highlighting an additional mechanism impacted by these gene mutations beyond what is impacted by the differentially expressed genes (Table S8).

### 3.3. Pediatric Cancers Harbor Mutations in PID-Related Genes

**Figure 3:**
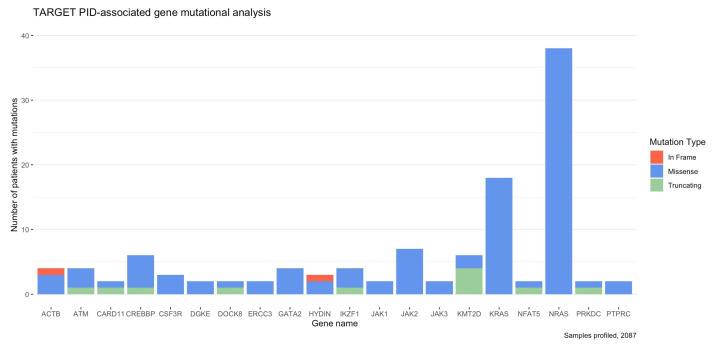
Mutation frequency of PID-related genes that are mutated in at least two of 2087 pediatric cancer patients profiled in AML, ALL, WT, RT, or NBL datasets. Classification of each mutation as being missense (blue), truncating (green), or in frame (red) is indicated in the stacked bar graph. Mutation profile was obtained from cBioPortal.

Mutational analysis of the five pediatric tissues using mutation data from the TARGET database revealed that out of the 472 PID-related genes, 64 were mutated in at least one of the 2087 patients, and 20 were mutated in two or more patients, resulting in a total of 115 different mutations observed in genes that were mutated in at least two patients (Figure 3). The top four mutated genes in pediatric cancers were *NRAS* (1.8%), *KRAS* (0.9%), *JAK2* (0.3%), and *CREBBP* (0.3%), highlighting that these genes should be analyzed further to assess the clinical significance of these mutations (Figure 3).

The most common mutations observed were missense mutations (88.7%), followed by truncating mutations (9.6%) and in-frame deletion mutations (1.7%; Figure 3). Genes that included multiple patients with the same mutation were *CREBBP* (R1446C), *CSF3R* (T618I), *JAK2* (R683S and D873N), *KRAS* (G12D, G13D, and G12V), and *NRAS* (Q61K, Q61R, G12D, G13D, G12S, and G12A; Table 1). These mutations highlight the need to further assess their clinical significance in pediatric cancers.

### 3.4. Copy Number Alterations in PID-Related Genes among Pediatric Cancers

**Figure 4:**
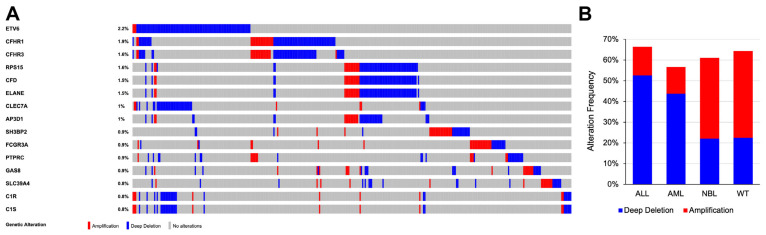
Copy number variation in PID-related genes among pediatric cancer patients. (**A**) Oncoprint depicting the major copy number alterations observed in pediatric ALL, AML, NBL, and WT samples. (**B**) Overall copy number alteration frequencies of PID-related genes with amplification and deletion status in each pediatric cancer cohort. Data were obtained from TARGET datasets on cBioPortal (v3.7.20).

Copy number variation (CNV) analysis of four TARGET cancer cohorts (ALL, AML, NBL, and WT) resulted in the identification of 4865 copy number alterations (amplified/deleted regions) across 1105 patients (Table S4). Significant focal amplifications in the genes *PNP*, *FCGR3A*, and *CFHR1* were found in a small fraction of the patients (Figure 4A), while deep deletions were observed in *ETV6*, *CFHR1*, and *RPS15*, especially for pediatric ALL and AML (Figure 4A). Moreover, pediatric ALL and AML were most affected by deep deletions in PID-related genes, with an overall alteration frequency of 66% and 57% across patients in their respective cancer cohorts and deep deletions making up 52% and 44% of the overall frequency, respectively (Figure 4B). Pediatric WT had an overall alteration frequency of 64%, the majority of which were gene amplifications (42%; Figure 4B). In addition, 61% of pediatric NBL patients harbored copy number alterations, where 39% were amplifications and 22% were deep deletions (Figure 4B). The overall alteration frequencies were determined by dividing the number of PID-related genes altered by the total number of profiled patients in each cancer cohort.

### 3.5. Altered PID-Related Genes Affect Overall Survival in Pediatric Cancer Cohorts

Survival analysis of the panel of PID-related genes in all TARGET cohorts identified a significant difference in overall survival, with the altered group having significantly lower survival (*p* = 0.0250; Figure S4). Survival analysis of genes mutated in two or more patients also revealed significant differences in overall survival, with the altered group having significantly decreased survival (*p* = 1.724 × 10^−6^; Figure S4).

To better assess the implications of the direction of differential expression on prognosis, a survival analysis was also conducted on each significant PID-related DEG for each cancer type. Significant differences in overall survival were determined using Cox regression and Kaplan–Meier survival analysis. A total of 79 genes were found to have significant effects on prognosis based on their expression (Table S5), and 23 genes had significant impacts on prognosis in at least two different cancer types, including *C1R*, *RPL5*, and *TERT*. Higher expressions of *C1R* and *RPL5* both had significant impacts on overall survival for ALL, AML, and RT, whereas high expression of *TERT* was significant for survival in ALL, AML, and NBL (Table S5). The results from STRING when assessing the identified GO enrichment of biological processes of the genes are described (Figure S5 and Table S6). 

**Figure 5:**
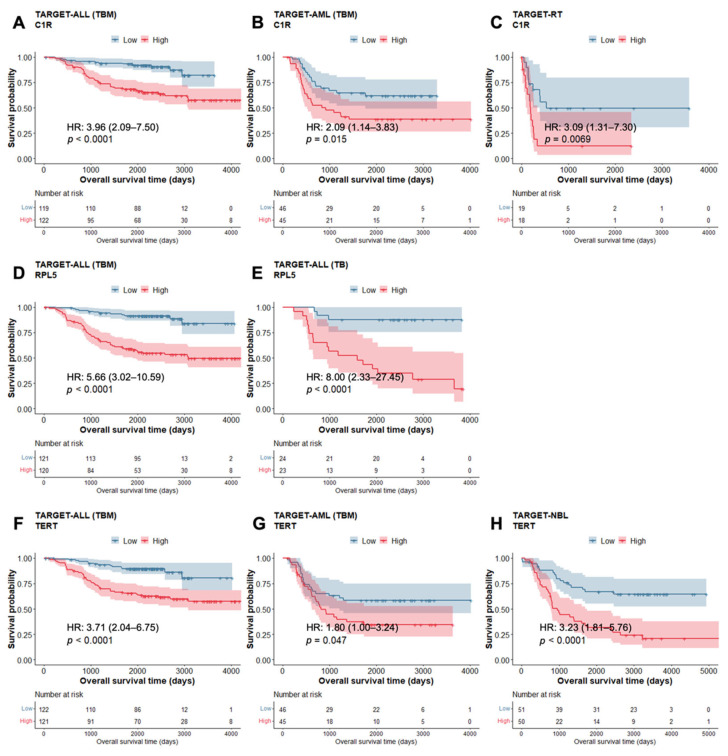
Kaplan–Meier survival curves for *C1R*: (**A**) ALL (TBM), (**B**) AML (TBM), and (**C**) RT, for *RPL5*: (**D**) ALL (TBM), (**E**) ALL (TB), and for *TERT*: (**F**) ALL (TBM), (**G**) AML (TBM), and (**H**) NBL. Cox proportional hazard ratios (HR) and 95% CI are described. *p*-value is based on log-rank test and 95% CI is represented by the shaded region.

For all cancer types, upregulation of the *C1R* gene was associated with poor prognosis in pediatric cancer patients (Figure 5), especially in the ALL cohort (Figure 5A). For ALL, upregulation of the *RPL5* gene was associated with decreased overall survival (Figure 5). Note that ALL produced the most significant results from this analysis (Figure 5D,E). Kaplan–Meier curves generated for *TERT* demonstrate that for ALL, AML, and NBL cancer types, upregulation of *TERT* results in poorer prognoses (Figure 5). Interestingly, ALL and NBL had the most significant differences in overall survival based on the increased expression of *TERT* (Figure 5F,H).

## 4. Discussion

Differential expression analysis with the *limma-voom* pipeline highlighted multiple genes, *CR2, MS4A1,* and *SEMA3A*, which should be further analyzed, given their prevalence among multiple cancer types. *CR2* encodes complement receptor 2 (CD21) and is a co-receptor on B cells, which play a central role in autoimmunity, such that patients with systemic lupus erythematosus show reduced CR2 levels [20]. CR2 also plays a role in recognizing foreign DNA during immune defense responses, which may indicate that CR2 contributes to the immune surveillance of tumor cells [3,20]. *MS4A1* encodes CD20, which is known to be induced by chemokine signaling and has been shown to interact with multiple B-cell surface proteins [21]. Interestingly, CD20 is expressed on the surface of both normal and malignant B-cells and has been a target for therapies using anti-CD20 monoclonal antibodies, highlighting that these therapies may also be effective for pediatric cancers [21]. Patients with Kallmann syndrome who experience gonadotropin-releasing hormone deficiency have been found to exhibit mutations in *SEMA3A*; however, regarding immune function, *SEMA3A* specifically has been found to act as an immune cell over-activation suppressor and is downregulated in some autoimmune diseases [22,23]. The aforementioned genes highlight molecular mechanisms, including B-cell receptor signaling and the suppression of immune cell activation, which are important immune functions that should be studied further to better understand the pathways between PIDs and the development of pediatric cancers. 

Differential expression analysis highlighted two additional genes, *RAG1* and *IGLL1,* for further study due to their identification in both DEA pipelines. RAG1 is responsible for the initiation of V(D)J recombination, which diversifies and strengthens the antigen-specific receptors of T-cells and B-cells, and mutations in the gene have been associated with immunodeficient and autoimmune clinical phenotypes [24]. Recent studies have shown that the high expression of *RAG1* is a potential driver of oncogenesis in B-cell ALL, and the aberrant activity of RAG1/2 is associated with the promotion of lymphocytic malignancies due to chromosomal translocations and DNA deletions in cancer genes [25,26]. Mutations in *IGLL1* have been observed to lead to defects in B-cell function, as it belongs to the immunoglobulin gene superfamily and is important in humoral immunity [27]. Genes that are most commonly differentially expressed between normal and cancer tissues are often involved in B-cell receptor regulation or signaling, indicating an important molecular mechanism requiring further exploration to increase our understanding of the relationship between PIDs and pediatric cancers.

Protein–protein network interactions identified through STRING analysis revealed several biological connections between the proteins translated by PID-related DEGs and genes mutated in two or more pediatric cancer patients. Given our developing understanding of the impact of PID-related genes on malignancies, the common theme of immune system processes being widely involved was expected. However, enrichment analysis has identified additional pathways in the mutated PID-related genes that could demonstrate the potential of these genes to alter cell cycle progression and metastasis, which are hallmarks of cancer, due to the enrichment of proliferative and motility processes [3,4,5]. Further analysis is necessary to outline how these mutations can lead to the development and progression of pediatric cancers.

Leukemia and PIDs are both disorders of the hematopoietic system [3,28]. Therefore, our findings were consistent with our expectations: PID-related genes were more commonly differentially expressed in pediatric leukemias and produced meaningful enrichment analysis results because similar immune components were likely involved. To better understand the molecular mechanisms linking PIDs to pediatric leukemias, differentially expressed PID-related genes and altered pathways in pediatric leukemias should be further analyzed. 

The mutational analysis highlighted the likely importance of *NRAS*, *KRAS*, *JAK2*, and *CREBBP* in pediatric cancer development because these genes are the most commonly mutated genes in pediatric cancer patients. Mutations in the *RAS* genes, including *NRAS* and *KRAS*, have been observed in many tumors, with *NRAS* mutations being more common in patients with AML [29]. The promotion of oncogenesis has been observed as a result of mutations in Ras proteins, highlighting the importance of further studying these genes, given their role in pediatric cancer molecular mechanisms and development [30]. *JAK2* mutations have been observed in malignancies and hematologic diseases [31]. *JAK2* encodes a cytoplasmic tyrosine kinase that is important in the signal transduction of hematopoietic growth factors, which is necessary to maintain the integrity of cellular viability [31,32]. *CREBBP* is involved in histone modification [33]. Interestingly, alterations in CREBBP among pediatric patients with ALL who do not relapse are rare, but *CREBBP* mutations are common in childhood ALL cases that do relapse, highlighting the importance of further elucidating their role in pediatric cancers [33]. 

The mutational analysis also highlighted specific mutations found in multiple pediatric cancer patients. The CREBBP R1446 position is located in the histone acetyltransferase domain, which is involved in substrate binding [33]. The R1446 location is noted to be a hotspot for driver mutations associated with pediatric ALL, with missense mutations at R1446C, R1446H, and others being common [33,34]. *CSF3R* mutations were previously identified as oncogenic, and the T618I mutation has been identified to truncate the cytoplasmic tail of CSF3R, which is similar to *CSF3R* mutations that have been observed to progress congenital neutropenia to AML [35]. Mutations involved in the amino acid residue R683 of *JAK2* are commonly found in childhood cancers and impact the auto-regulation of JAK2 activity [36]. Specifically, R683S has been identified in ALL, and it may upregulate phosphorylation levels and the activation of the MEK/ERK pathway [36,37]. The MEK/ERK pathway is necessary to break B-cell tolerance, indicating a possible mechanism that can explain how the R683S mutation may lead to the development of PIDs and pediatric cancers [38]. Mutation D873N of *JAK2* has also been linked to ALL [37]. JAK2 is a signaling molecule for cytokines; thus, when mutated, it may indicate a mechanism explaining altered immune surveillance in pediatric cancers [39]. *KRAS* mutations at the amino acid location G12 have been associated with more aggressive cancer types and a poorer prognosis [40]. *KRAS* G13 mutations have also been identified in cancers and have been potential therapeutic targets for epidermal growth factor receptor (EGFR) inhibitors [41]. All *NRAS* mutations at amino acid locations G12, G13, and Q61 have been identified in cancers and upregulate NRAS cycling; however, they impact NRAS activity differently [42,43]. The incidence of these mutations varies among cancer types such that *NRAS* mutations in melanomas are almost entirely at the Q61 position [43]. All mutations observed in at least two different patient profiles had previously been identified in the literature on cancer; however, they have been found to alter different mechanisms. In addition, the extent of knowledge of the aforementioned mutations varies. Therefore, the pathways that connect these genes and the specific molecular mechanisms impacted must be further analyzed to elucidate the relationship between PID-related genes and pediatric cancers. Despite the specific mutations that differ between PIDs and pediatric cancers, there are common PID-related genes that are mutated in pediatric cancers, highlighting their likely relationship. 

CNV analysis revealed that a significant proportion of PID-related genes were altered across the four pediatric cancer cohorts examined. Deep deletion of *ETV6*, a transcription-regulating gene, has been previously implicated in leukemias and may play a role in oncogenesis, treatment response, and outcome in pediatric ALL patients [44,45,46,47]. Furthermore, the analysis identified CNVs in the ribosomal protein (RP) gene family, specifically *RPS15*, with relation to deep deletion in pediatric ALL and AML. In a study of adult cancers, a significant prevalence of alteration in RP genes was observed, with 82% of tumors harboring single/double deletions within these genes [48]. 

CNVs in the complement factor H (*CFH*) gene cluster were commonly identified in the CNV analysis, with *CFHR1* being notably deep deleted in the pediatric ALL and AML samples. Interestingly, a high prevalence of deletions in *CFH*-related genes 3 and 1 (*CFHR3-CFHR1*) have been detected in pediatric cancer patients post-hematopoietic stem cell transplant (HSCT) [49]. Heterozygous deletions in *CFHR3-CFHR1* genes have also been linked to atypical hemolytic uremic syndrome in pediatric ALL patients and transplant-associated thrombotic microangiopathy in neuroblastoma patients [50,51]. As such, the role of CNVs in PID-related genes and their significance in the development, maintenance, and outcomes of pediatric cancers should be thoroughly considered.

Only three genes—*ACTB*, *CSF3R*, and *GATA2*—were identified in both the DEA and mutational analysis, which highlights their possible significance in cancer and disease development. Mutations in *ACTB* have been reported in congenital developmental disorders and adult lymphoid hematological cancers as well as pericytomas when an *ACTB* gene fusion is present [52,53,54]. However, little has been reported on its role in pediatric cancers. *CSF3R* encodes the receptor for colony-stimulating factor 3, which is a member of the IL-6 superfamily of cytokines, and mutations in the gene have led to the development of AML [35]. *GATA2* variants carry a known predisposition to pediatric cancers due to the disruption of the maintenance of hematopoietic stem cells [55]. The three commonly altered genes play roles in important mechanisms regarding pediatric cancer development. However, the interplay between them should be further analyzed to better understand the implications when they are dysregulated. For instance, these variants of interest may be investigated for their impact on oncogenesis and immune evasion through comparative screening of critical oncogenic and immune-related markers in altered and unaltered cells, or qualitative analysis of tissue for such markers in affected and unaffected patients. 

Using cBioPortal, significant differences in prognosis were identified for the panel of PID-related genes and genes mutated in two or more patients. Therefore, variants in PID-related genes may have an impact on the prognosis of those with pediatric cancers, and the mechanisms behind how the mutations lead to poorer outcomes should be studied. Furthermore, this also highlights the poorer prognosis for individuals with variant expression in the commonly mutated genes, which may indicate that alternative clinical approaches are needed for these patients to improve their prognosis. Continued analysis regarding survival outcomes of patients based on the direction of differential expression should be conducted to better understand the implications of altered regulations of all the identified genes. 

Survival analysis of each significant PID-related DEG in each cancer type identified significant differences in prognosis based on the regulation of many genes. *C1R*, which was identified to decrease overall survival for patients with ALL, AML, and RT when upregulated, has been reported to promote the progression of squamous cell carcinoma when elevated [56]. The findings suggested that *C1R* may be a common prognostic indicator for a variety of cancer types. *RPL5* has been noted to be a tumor suppressor gene, and downregulation of the gene has been associated with cancer recurrence and poor prognosis [57,58]. This highlights the likely reason for the downregulation of *RPL5* resulting in poorer prognosis for RT patients but not the reason for the upregulation of RPL5 resulting in poorer prognosis for both ALL and AML patients. Further analysis is necessary to understand the differences in RPL5 mechanisms of action in leukemias compared to other cancer types regarding its role in prognosis. 

Upregulation of *TERT* was determined to worsen the patient prognosis for multiple cancer types, which may be expected based on reports that telomerase activity increases with upregulation of *TERT* [59]. Increased telomerase activity allows for greater proliferative potential and immortality of cancerous cells, which represent hallmarks of cancer [4,5]. Upregulation of TERT resulting in increased telomerase activity may, in part, explain the poor prognoses identified in this study and previously in breast cancer [59]. Identification of these genes that resulted in a poor prognosis for multiple cancer types indicated potential biomarkers that suggest the need to adapt treatment strategies from those currently used to improve patient outcomes.

Interestingly, the common DEGs had cancer-type selective results on prognosis. *MS4A1*, *CR2*, *IGLL1*, and *RAG1* each significantly impacted prognosis in ALL, and *SEMA3A* significantly impacted survival in AML. Upregulation of *MS4A1*, *IGLL1*, *RAG1*, and *SEMA3A* resulted in significantly decreased survival in pediatric cancer patients. Of particular note, *SEMA3A* was under-expressed in all identified cancer types compared to their healthy tissue samples, which may suggest that reactivation of *SEMA3A* is critical for cancer progression. In fact, a previous study indicated that *SEMA3A* upregulation promotes tumorigenesis and leads to poorer overall prognosis in adult cancers [60]. Considering that upregulation of *MS4A1*, *IGLL1*, and *RAG1* results in decreased overall survival, further analysis to better understand the implications of upregulation could highlight potential patterns for variations in prognosis. In contrast to the four genes highlighted through DEA, *CR2* was under-expressed in ALL in the DEA and resulted in a poorer prognosis in ALL. Therefore, further analysis of *CR2* regarding the mechanisms impacted by its downregulation should be performed to better determine its prognostic impact and may suggest the need for alternative therapeutic approaches to be pursued for individuals with pediatric ALL and *CR2* downregulation. 

Multiple significant DEGs were observed, including *CR2*, *MS4A1*, *IGLL1*, *RAG1*, and *SEMA3.* Many DEGs were involved in B-cell receptor regulation and signaling, highlighting pathways that must be further explored to assess their significance in pediatric cancer development and outcomes. Enrichment analysis indicated that pediatric leukemias were most commonly involved in significantly altered molecular pathways and PID-related gene expression. While this may be expected based on the hematological relationship between leukemia and PIDs, the mechanisms behind this relationship should be further explored. 

Protein–protein interactions and GO pathway analysis further confirmed these findings, in addition to highlighting biological processes related to hallmarks of cancer and may suggest some of the mechanisms of action by which the identified PID-related genes impact cancer development. The mutational analysis determined that genes previously identified in cancers were most commonly observed in the profiled patients with mutations in *NRAS*, *KRAS*, *JAK2*, and *CREBBP*. Additionally, CNV analysis identified amplification of *PNP*, *FCGR3A*, and *CFHR1* as well as deep deletion of *ETV6*, *CFHR1*, and *RPS15* as the most frequently detected copy number altered PID-related genes across the four available pediatric cancer cohorts. Multiple PID-related genes appear to have significant implications in pediatric cancers; however, the distinct molecular mechanisms between PIDs and pediatric cancers in relation to these genes must be further explored.

Overall survival was decreased for the PID-related and commonly mutated genes, which highlights the need for further investigation into these genes to better understand the mechanisms of action being altered and their potential clinical significance when treating patients. Furthermore, survival analysis of each significant PID-related DEG determined that *C1R, RPL5*, and *TERT* may be indicative of patient prognosis when differentially expressed and should be studied further to better understand how treatment alterations regarding these genes may improve patient outcomes.

## 5. Conclusions

Future directions from this project include investigating differences in expression and patient prognosis based on smaller subsets of age groups including children, youth, and young adults as well as additional cancer types. This study highlighted multiple PID-related genes for further investigation regarding their implications in PIDs and pediatric cancer mechanisms, which may lead to the identification of new therapeutic targets.

## Figures and Tables

**Table 1:** Summary of protein changes for 64 PID-related genes identified in patients with the following cancer types: ALL, AML, NBL, RT, and WT. All data were extracted individually for each cancer type on cBioPortal and then summarized.

Gene Mutated	Cancer Type	No. of Patients	Mutation Type	Protein Change	Avg. Allele Freq.	OncoKB Status
ACTB	ALL	1	MISSENSE	E205K	0.36	
	WT	1	MISSENSE	R147L	0.35	
	WT	1	IF DEL	I282del	0.34	
	WT	1	MISSENSE	G146V	0.55	
ADAM17	ALL	1	MISSENSE	C506F	0.38	
ATM	ALL	1	MISSENSE	Y1248H	0.48	
	NBL	1	NONSENSE	Q3038*	0.49	LO
	NBL	1	MISSENSE	A911S	0.35	
C5	WT	1	MISSENSE	V1108I	0.28	
CARD11	NBL	1	MISSENSE	Y963C	0.25	
	NBL	1	SPLICE	X1007_splice	0.40	
CARD9	AML	1	MISSENSE	L54M	0.42	
CREBBP	ALL	3	MISSENSE	R1446C	0.65	LO
	ALL	1	MISSENSE	R1446H	0.11	LO
	ALL	1	NONSENSE	R1360*	0.40	LO
	ALL	1	MISSENSE	P1494A	0.34	
CSF3R	ALL	1	MISSENSE	T618I	0.55	O
	AML	2	MISSENSE	T618I	0.29	O
CXCR4	AML	1	NONSENSE	R334*	0.30	LO
DGKE	ALL	1	MISSENSE	M123T	0.41	
	WT	1	MISSENSE	E460G	0.11	
DNAAF2	AML	1	MISSENSE	V141I	0.67	
DNAAF3	WT	1	MISSENSE	E292G	0.50	
DNAH1	ALL	1	MISSENSE	S3418P	0.69	
DNAH11	ALL	1	MISSENSE	S3079L	0.17	
DNAH5	WT	1	MISSENSE	T179A	0.09	
DOCK8	NBL	1	MISSENSE	P711T	0.29	
	NBL	1	SPLICE	X179_splice	0.65	
ERCC3	ALL	1	MISSENSE	G130E	0.55	
	AML	1	MISSENSE	I220S	0.46	
ETV6	AML	1	MISSENSE	F102L	0.20	
	AML	1	MISSENSE	R103G	0.20	
F7	WT	1	MISSENSE	Y104H	0.16	
FANCA	ALL	1	MISSENSE	V6D	0.59	
FANCE	AML	1	MISSENSE	S52G	0.39	
FANCM	NBL	1	MISSENSE	E1744K	0.33	
FAT4	WT	1	MISSENSE	K4272N	0.32	
GAS8	AML	1	MISSENSE	R369H	0.26	
GATA1	AML	1	MISSENSE	P73L	0.37	
GATA2	AML	1	MISSENSE	L321P	0.16	
	AML	1	MISSENSE	N317I	0.28	
	AML	1	MISSENSE	A318V	0.35	
	AML	1	MISSENSE	A318T	0.21	LN
	AML	1	MISSENSE	R396Q	0.45	
HYDIN	WT	1	MISSENSE	T2521P	0.36	
	WT	1	MISSENSE	K2518R	0.08	
	WT	1	IF DEL	T2521_R2525del	0.26	
IKBKB	ALL	1	MISSENSE	N109S	0.21	
IKZF1	AML	1	MISSENSE	R502W	0.42	
	AML	1	MISSENSE	C175Y	0.52	
	AML	1	MISSENSE	N159S	0.33	
	AML	1	NONSENSE	K380*	0.42	
IL7R	ALL	1	MISSENSE	S185C	0.31	O
JAK1	ALL	1	MISSENSE	V658F	0.26	O
	ALL	1	MISSENSE	Q572L	0.16	
JAK2	ALL	1	MISSENSE	R683G	0.33	O
	ALL	3	MISSENSE	R683S	0.20	O
	ALL	2	MISSENSE	D873N	0.30	
	AML	1	MISSENSE	V617F	0.17	O
	AML	1	MISSENSE	M535I	0.20	LO
JAK3	AML	1	MISSENSE	M511I	0.14	O
	AML	1	MISSENSE	L857P	0.18	LO
KDM6A	AML	1	MISSENSE	R1111P	0.28	
KMT2D	ALL	1	MISSENSE	R5432W	0.20	LO
	ALL	1	FS INS	K287Ffs*2	N/A	LO
	ALL	1	FS DEL	Q170Afs*49	0.31	LO
	WT	1	MISSENSE	I1336V	0.48	
	AML	1	NONSENSE	Q2416*	0.44	LO
	AML	1	NONSENSE	R4960*	0.21	LO
	AML	1	MISSENSE	G5410E	0.47	
KRAS	ALL	2	MISSENSE	G13D	0.36	
	ALL	1	MISSENSE	G12S	0.12	
	ALL	4	MISSENSE	G12D	0.33	O
	ALL	1	MISSENSE	G12V	0.41	O
	WT	1	MISSENSE	G12D	0.47	O
	AML	1	MISSENSE	Q61H	0.46	O
	AML	2	MISSENSE	G13D	0.26	O
	AML	2	MISSENSE	G12V	0.38	O
	AML	3	MISSENSE	G12D	0.49	O
	AML	1	MISSENSE	K117N	0.26	O
MLPH	ALL	1	MISSENSE	P429L	0.28	
MSN	WT	1	MISSENSE	E41G	0.14	
NBN	RT	1	MISENSE	Q291H	N/A	
NFAT5	WT	1	NONSENSE	Q36*	0.44	
	NBL	1	MISSENSE	L390M	0.31	
NFKB1	WT	1	MISSENSE	D544G	0.09	
NLRC4	ALL	1	MISSENSE	D863G	0.45	
NLRP12	WT	1	MISSENSE	R138W	0.56	
NLRP3	NBL	1	MISSENSE	R141I	0.36	
NRAS	ALL	6	MISSENSE	G12D		O
	ALL	1	MISSENSE	Q61K	0.41	O
	ALL	1	MISSENSE	Q61P	0.28	LO
	AML	1	MISSENSE	G12S	0.15	O
	AML	5	MISSENSE	G13D	0.42	O
	AML	1	MISSENSE	G13V	0.44	O
	AML	1	MISSENSE	G13R	0.34	O
	AML	3	MISSENSE	Q61R	0.37	O
	AML	9	MISSENSE	Q61K	0.33	O
	AML	2	MISSENSE	G12D	0.22	O
PCCA	ALL	1	MISSENSE	D458H	0.42	
POLE	WT	1	MISSENSE	R924C	0.30	
PRKDC	WT	1	MISSENSE	R2914H	0.54	
	NBL	1	SPLICE	X259_splice	0.61	
PTPRC	NBL	1	MISSENSE	P290S	0.39	
	NBL	1	MISSENSE	N176S	0.38	
RAC2	AML	1	MISSENSE	G12R	0.21	
RAD50	WT	1	MISSENSE	T1054A	0.92	
RNASEH2B	WT	1	MISSENSE	E168G	0.28	
RNF168	ALL	1	MISSENSE	N498D	0.33	
RNF31	WT	1	MISSENSE	L302F	0.49	
RORC	NBL	1	MISSENSE	L267M	0.33	
SLC39A4	AML	1	MISSENSE	L561M	0.51	
SPINK5	ALL	1	MISSENSE	R790Q	0.47	
STIM1	ALL	1	MISSENSE	C49W	0.28	
STK4	ALL	1	NONSENSE	W99*	0.36	
TCF3	ALL	1	FS DEL	G470Afs*16	0.43	LO
	ALL	1	MISSENSE	H460Y	0.46	
TERT	WT	1	MISSENSE	Q829R	0.10	
TTC37	WT	1	MISSENSE	R1296S	0.58	
TYK2	AML	1	MISSENSE	V678L	0.24	
ZAP70	ALL	1	MISSENSE	R360C	0.55	

Abbreviations: LN, likely neutral; LO, likely oncogenic; O, oncogenic; R, resistance. * indicates truncation/stop codon in the protein sequence, which is common nomenclature.

## Data Availability

The code used in this project is available at https://github.com/shaelenestanding/NarendranLab (accessed on 21 July 2022). The results published here are in whole or part based upon data generated by the Therapeutically Applicable Research to Generate Effective Treatments (TARGET) initiative, phs000218. The data used for this analysis are available at https://portal.gdc.cancer.gov/projects (accessed on 21 July 2022).

## Supplementary Materials

The following supporting information can be downloaded at: https://www.mdpi.com/article/10.3390/cancers14235942/s1, Figure S1: EnhancedVolcano plots produced from the DEA of PID-related genes for the (A) ALL-P1 (TBM), (B) ALL-P1 (TB), (C) ALL-P2 (TBM), (D) ALL-P2 (TB), (E) AML (TBM), (F) AML (TB), (G) CCSK, (H) NBL, (I) RT, and (J) WT TARGET tumor samples against the GTEx-blood or kidney normal tissue samples depending on cancer type. This analysis was performed with the TCGAbiolinks R package using the limma-voom pipeline. Figure S2: EnhancedVolcano plots produced from the DEA of PID genes for the (A) ALL-P1 (TBM), (B) ALL-P1 (TB), (C) ALL-P2 (TBM), (D) ALL-P2 (TB), (E) AML (TBM), (F) AML (TB), (G) CCSK, (H) NBL, (I) RT, and (J) WT TARGET tumor samples against the GTEx-blood or kidney normal tissue tissues depending on cancer type. This analysis was performed with the TCGAbiolinks R package using the edgeR pipeline. Figure S3: Venn diagram summary of DEA results for PID-related genes that are significantly differentially expressed in pediatric cancer types including AML, ALL, WT, RT, and NBL. The different gene names are listed in each of the cancer types in which they were significantly differentially expressed in. The colour of the gene represents the differential expression of each gene either upregulated (red), downregulated (blue), or mixed (black) if the gene had mixed regulation in different cancer types. The analyses have been performed using the TCGAbiolinks R package and the limma-voom pipeline. Figure S4: Kaplan-Meier survival curve summary for (A) 472 PID-related genes (*p* = 0.025) and (B) 20 genes mutated in >0.1% of patients (*p* = 1.724 × 10^−6^). The altered group is defined as harboring somatic mutations from whole genome or whole exome sequencing. Only patients with mutations data were used from the portal in this analysis. *p* < 0.05 was considered to indicate statistical significance. Analysis was done using cBioPortal including the following TARGET projects: ALL-P2, AML, NBL, RT, and WT. Figure S5: STRING network visualization retrieved for the 23 PID-related genes with significant effects on overall survival in at least two different pediatric cancer types. STRING (v11) online platform was used for analysis. Figure S6: STRING network visualization retrieved for the 89 PID-related differentially expressed genes in pediatric cancers. STRING (v11) online platform was used for analysis. Figure S7: STRING network visualization retrieved for the 20 PID-related genes mutated in two or more pediatric cancer patients. STRING (v11) online platform was used for analysis. Table S1: Summary of DEA results for PID-related genes that are significantly differentially expressed in pediatric cancer types including AML, ALL, WT, RT, and NBL. Analyses were performed using the TCGAbiolinks R package and the limma-voom (use adj. p-value) or edgeR (use FDR) pipeline. Table S2: GO ID and terms from the biological process GO enrichment analysis of all differentially expressed genes TARGET tumor samples against the GTEx normal tissue samples. This analysis was performed with the topGO R package. Table S3: GO ID and terms from the biological process GO enrichment analysis of all PID-related differentially expressed genes TARGET tumor samples against the GTEx normal tissue samples. This analysis was performed with the topGO R package. Table S4: Summary copy number variant analysis for 472 PID-related genes in all the following cancer types: ALL, AML, NBL and WT. Attached file: Supplementary_Data_S1.xlsx. Table S5: Summary survival analysis results for 89 differentially expressed genes in the following cancer types: ALL, AML, CCSK, NBL, RT, and WT. This analysis was done using Cox regression analysis with a generated R script. Attached file: Supplementary_Data_S2.xlsx. Table S6: Summary results from the biological process GO enrichment analysis of the 23 PID-related genes with significant effects on overall survival in at least two different pediatric cancer types. This analysis was performed using STRING (v11). Table S7: Summary results from the biological process GO enrichment analysis of the 89 PID-related differentially expressed genes. Only the top 40 terms are included in this table. This analysis was performed using STRING (v11). Table S8: Summary results from the biological process GO enrichment analysis of the 20 PID-related genes mutated in two or more pediatric cancer patients. Only the top 40 terms are included in this table. This analysis was done using STRING (v11).

## References

[1] Canadian Cancer Society’s Advisory Committee on Cancer Statistics (2015). Canadian Cancer Statistics 2015.

[2] Derpoorter C., Bordon V., Laureys G., Haerynck F., Lammens T. (2018). Genes at the Crossroad of Primary Immunodeficiencies and Cancer. Front. Immunol..

[3] Hauck F., Voss R., Urban C., Seidel M.G. (2018). Intrinsic and extrinsic causes of malignancies in patients with primary immunodeficiency disorders. J. Allergy Clin. Immunol..

[4] Hanahan D., Weinberg R.A. (2000). The Hallmarks of Cancer. Cell.

[5] Hanahan D., Weinberg R.A. (2011). Hallmarks of cancer: The next generation. Cell.

[6] Tangye S.G., Al-Herz W., Bousfiha A., Cunningham-Rundles C., Franco J.L., Holland S.M., Klein C., Morio T., Oksenhendler E., Picard C. (2022). Human inborn errors of immunity: 2022 update on the classification from the International Union of Immunological Societies Expert Committee. J. Clin. Immunol..

[7] (2020). Proceedings of the Canadian Society of Allergy and Clinical Immunology Annual Scientific Meeting 2019. Allergy Asthma Clin Immunol..

[8] (2021). Proceedings of the Canadian Society of Allergy and Clinical Immunology Annual Scientific Meeting 2020. Allergy Asthma Clin. Immunol..

[9] Mounir M., Lucchetta M., Silva T.C., Olsen C., Bontempi G., Chen X., Noushmehr H., Colaprico A., Papaleo E. (2019). New functionalities in the TCGAbiolinks package for the study and integration of cancer data from GDC and GTEx. PLoS Comput. Biol..

[10] Grossman R.L., Heath A.P., Ferretti V., Varmus H.E., Lowy D.R., Kibbe W.A., Staudt L.M. (2016). Toward a Shared Vision for Cancer Genomic Data. N. Engl. J. Med..

[11] Collado-Torres L., Nellore A., Kammers K., Ellis S.E., Taub M.A., Hansen K.D., Jaffe A.E., Langmead B., Leek J.T. (2017). Reproducible RNA-seq analysis using recount2. Nat. Biotechnol..

[12] Bartha Á., Győrffy B. (2021). TNMplot.com: A Web Tool for the Comparison of Gene Expression in Normal, Tumor and Metastatic Tissues. Int. J. Mol. Sci..

[13] Zeng W.Z.D., Glicksberg B.S., Li Y., Chen B. (2019). Selecting precise reference normal tissue samples for cancer research using a deep learning approach. BMC Med. Genom..

[14] Ritchie M.E., Belinda P., Wu D., Hu Y., Law C.W., Shi W., Smyth G.K. (2015). limma powers differential expression analyses for RNA-sequencing and microarray studies. Nucleic Acids Res..

[15] Lucchetta M., Da Piedade I., Mounir M., Vabistsevits M., Terkelsen T., Papaleo E. (2019). Distinct signatures of lung cancer types: Aberrant mucin O-glycosylation and compromised immune response. BMC Cancer.

[16] Comprehensive Primary Immunodeficiency NGS Panel Fulgent Genetics. https://www.fulgentgenetics.com/Comprehensive-Primary-Immunodeficiency.

[17] Gao J., Aksoy B.A., Dogrusoz U., Dresdner G., Gross B.E., Sumer S.O., Sun Y., Jacobsen A., Sinha R., Larsson E. (2013). Integrative Analysis of Complex Cancer Genomics and Clinical Profiles Using the cBioPortal. Sci. Signal..

[18] Alexa A., Rahnenführer J. (2009). Gene set enrichment analysis with topGO. Bioconduct. Improv..

[19] Szklarczyk D., Gable A.L., Lyon D., Junge A., Wyder S., Huerta-Cepas J., Simonovic M., Doncheva N.T., Morris H.J., Bork P. (2019). STRING v11: Protein-protein association networks with increased coverage, supporting functional discovery in genome-wide experimental datasets. Nucleic Acids Res..

[20] Asokan R., Banda N.K., Szakonyi G., Chen X.S., Holers V.M. (2013). Human complement receptor 2 (CR2/CD21) as a receptor for DNA: Implications for its roles in the immune response and the pathogenesis of systemic lupus erythematosus (SLE). Mol. Immunol..

[21] Pavlasova G., Mraz M. (2020). The regulation and function of CD20: An “enigma” of B-cell biology and targeted therapy. Haematologica.

[22] Hanchate N.K., Giacobini P., Lhuillier P., Parkash J., Espy C., Fouveaut C., Leroy C., Baron S., Campagne C., Vanacker C. (2012). SEMA3A, a Gene Involved in Axonal Pathfinding, Is Mutated in Patients with Kallmann Syndrome. PLoS Genet..

[23] Rezaeepoor M., Ganjalikhani-Hakemi M., Shapoori S., Eskandari N., Sharifi M., Etemadifar M., Marjan M. (2018). Semaphorin-3A as an immune modulator is suppressed by microRNA-145-5p. Cell J..

[24] Notarangelo L.D., Kim M.-S., Walter J.E., Lee Y.N. (2016). Human RAG mutations: Biochemistry and clinical implications. Nat. Rev. Immunol..

[25] Han Q., Ma J., Gu Y., Song H., Kapadia M., Kawasawa Y.I., Dovat S., Song C., Ge Z. (2019). RAG1 high expression associated with IKZF1 dysfunction in adult B-cell acute lymphoblastic leukemia. J. Cancer.

[26] Rommel P.C., Oliveira T.Y., Nussenzweig M.C., Robbiani D.F. (2017). RAG1/2 induces genomic insertions by mobilizing DNA into RAG1/2-independent breaks. J. Exp. Med..

[27] Luo X., Huang S., Luo S., Liao H., Wang Y., Deng X., Ma F., Ma C.W., Zhou L. (2018). Identification of genes underlying the enhancement of immunity by a formula of lentinan, pachymaran and tremelia polysaccharides in immunosuppressive mice. Sci. Rep..

[28] Whichard Z.L., Sarkar C.A., Kimmel M., Corey S.J. (2010). Hematopoiesis and its disorders: A systems biology approach. Blood.

[29] Wang S., Wu Z., Li T., Li Y., Wang W., Hao Q., Xie X., Wan D., Jiang Z., Wang C. (2020). Mutational spectrum and prognosis in NRAS-mutated acute myeloid leukemia. Sci. Rep..

[30] Prior I.A., Lewis P.D., Mattos C. (2012). A Comprehensive Survey of Ras Mutations in Cancer. Cancer Res.

[31] Freitas R.M.D., Santos M.D.O., Maranduba C.M.D.C. (2013). The JAK2 gene as a protagonist in chronic myeloproliferative neoplasms. Rev. Bras Hematol. Hemoter..

[32] Farrar W.L., Brini A.T., Harel-Bellan A., Korner M., Ferris D.K. (1990). Hematopoietic growth-factor signal transduction and regulation of gene expression. Immunol. Ser..

[33] Mullighan C.G., Zhang J., Kasper L.H., Lerach S., Payne-Turner D., Phillips L.A., Heatley S.L., Holmfeldt L., Collins-Underwood J.R., Ma J. (2011). CREBBP mutations in relapsed acute lymphoblastic leukaemia. Nature.

[34] Chang Y., Woessner D.W., Lin W., Chen T., Xu B., Fan Y., Haiyan T., Junmin P., Lawryn K., Churchman M.L. (2017). CREBBP histone acetyltransferase domain mutations result in dexamethasone resistance in B-progenitor acute lymphoblastic leukemia. Blood.

[35] Maxson J.E., Gotlib J., Pollyea D.A., Fleischman A.G., Agarwal A., Eide C.A., Bottomly D., Wilmot B., McWeeney S.K., Tognon C.E. (2013). Oncogenic CSF3R mutations in chronic neutrophilic leukemia and atypical CML. N. Engl. J. Med..

[36] Steeghs E.M., Jerchel I.S., de Goffau-Nobel W., Hoogkamer A.Q., Boer J.M., Boeree A., van de Ven C., Koudijs M.J., Besselink N.J., de Groot-Kruseman H.A. (2017). *JAK2* aberrations in childhood B-cell precursor acute lymphoblastic leukemia. Oncotarget.

[37] Shan Y., Gnanasambandan K., Ungureanu D., Kim E.T., Hammarén H., Yamashita K., Silvennoinen O., Shaw D.E., Hubbard S.R. (2014). Molecular basis for pseudokinase-dependent autoinhibition of JAK2 tyrosine kinase. Nat. Struct. Mol. Biol..

[38] Greaves S.A., Peterson J.N., Torres R.M., Pelanda R. (2018). Corrigendum: Activation of the MEK-ERK Pathway Is Necessary but Not Sufficient for Breaking Central B Cell Tolerance. Front. Immunol..

[39] Alabdulaali M.K. (2009). The role of JAK2 abnormalities in hematologic neoplasms. Hematol. Rep..

[40] Waters A.M., Der C.J. (2018). KRAS: The critical driver and therapeutic target for pancreatic cancer. Cold Spring Harb. Perspect. Med..

[41] Rabara D., Tran T.H., Dharmaiah S., Stephens R.M., McCormick F., Simanshu D.K., Holderfield M. (2019). KRAS G13D sensitivity to neurofibromin-mediated GTP hydrolysis. Proc. Natl. Acad. Sci. USA.

[42] Smith M.J., Neel B.G., Ikura M. (2013). NMR-based functional profiling of RASopathies and oncogenic RAS mutations. Proc. Natl. Acad. Sci. USA.

[43] Burd C.E., Liu W., Huynh M.V., Waqas M.A., Gillahan J.E., Clark K.S., Fu K., Martin B.L., Jeck W.R., Souroullas G.P. (2014). Mutation-Specific RAS Oncogenicity Explains NRAS Codon 61 Selection in Melanoma. Cancer Discov..

[44] Shen W., Szankasi P., Sederberg M., Schumacher J., Frizzell K.A., Gee E.P., Patel J.L., South S.T., Xu X., Kelley T.W. (2016). Concurrent detection of targeted copy number variants and mutations using a myeloid malignancy next generation sequencing panel allows comprehensive genetic analysis using a single testing strategy. Br. J. Haematol..

[45] Forero-Castro M., Robledo C., Benito R., Abáigar M., Martín A., Arefi M., Fuster J.L., Heras N.D.L., Rodríguez J.N., Quintero J. (2016). Genome-Wide DNA Copy Number Analysis of Acute Lymphoblastic Leukemia Identifies New Genetic Markers Associated with Clinical Outcome. PLoS ONE.

[46] Bokemeyer A., Eckert C., Meyr F., Koerner G., von Stackelberg A., Ullmann R., Türkmen S., Henze G., Seeger K. (2014). Copy number genome alterations are associated with treatment response and outcome in relapsed childhood ETV6/RUNX1-positive acute lymphoblastic leukemia. Haematologica.

[47] Järviaho T., Bang B., Zachariadis V., Taylan F., Moilanen J., Möttönen M., Smith C.I.E., Harila-Saari A., Niinimäki R., Nordgren A. (2019). Predisposition to childhood acute lymphoblastic leukemia caused by a constitutional translocation disrupting ETV6. Blood Adv..

[48] Panda A., Yadav A., Yeerna H., Singh A., Biehl M., Lux M., Schulz A., Klecha T., Doniach S., Khiabanian H. (2020). Tissue- and development-stage-specific mRNA and heterogeneous CNV signatures of human ribosomal proteins in normal and cancer samples. Nucleic Acids Res..

[49] Jodele S., Licht C., Goebel J., Dixon B.P., Zhang K., Sivakumaran T.A., Davies S.M., Pluthero F.G., Lu L., Laskin B.L. (2013). Abnormalities in the alternative pathway of complement in children with hematopoietic stem cell transplant-associated thrombotic microangiopathy. Blood.

[50] Cheng G., Ozgonenel B., Bhambhani K., Kapur G., Smith R., Savaşan S. (2018). Recurrent Atypical Hemolytic Uremic Syndrome in Children With Acute Lymphoblastic Leukemia Undergoing Maintenance Chemotherapy. J. Pediatr. Hematol..

[51] Nozawa A., Ozeki M., Hori T., Kawamoto N., Hirayama M., Azuma E., Fukao T. (2018). A Heterozygous CFHR3-CFHR1 Gene Deletion in a Pediatric Patient With Transplant-associated Thrombotic Microangiopathy Who was Treated with Eculizumab. J. Pediatr. Hematol..

[52] Witjes L., Van Troys M., Verhasselt B., Ampe C. (2020). Prevalence of Cytoplasmic Actin Mutations in Diffuse Large B-Cell Lymphoma and Multiple Myeloma: A Functional Assessment Based on Actin Three-Dimensional Structures. Int. J. Mol. Sci..

[53] Castro E., Cortes-Santiago N., Ferguson L.M.S., Rao P.H., Venkatramani R., López-Terrada D. (2016). Translocation t(7;12) as the sole chromosomal abnormality resulting in ACTB-GLI1 fusion in pediatric gastric pericytoma. Hum. Pathol..

[54] Kerr D.A., Pinto A., Subhawong T.K., Wilky B.A., Schlumbrecht M.P., Antonescu C.R., Nielsen G.P., Rosenberg A.E. (2019). Pericytoma with t(7;12) and ACTB-GLI1 fusion: Reevaluation of an unusual entity and its relationship to the spectrum of GLI1 fusion-related neoplasms. Am. J. Surg. Pathol..

[55] Bruzzese A., Leardini D., Masetti R., Strocchio L., Girardi K., Algeri M., Del Baldo G., Locatelli F., Mastronuzzi A. (2020). GATA2 Related Conditions and Predisposition to Pediatric Myelodysplastic Syndromes. Cancers.

[56] Riihilä P., Viiklepp K., Nissinen L., Farshchian M., Kallajoki M., Kivisaari A., Meri S., Peltonen J., Kähäri V., Peltonen S. (2019). Tumour-cell-derived complement components C1r and C1s promote growth of cutaneous squamous cell carcinoma. Br. J. Dermatol..

[57] Fancello L., Kampen K.R., Hofman I.J., Verbeeck J., De Keersmaecker K. (2017). The ribosomal protein gene RPL5 is a haploinsufficient tumor suppressor in multiple cancer types. Oncotarget.

[58] Dolezal J.M., Dash A.P., Prochownik E.V. (2018). Diagnostic and prognostic implications of ribosomal protein transcript expression patterns in human cancers. BMC Cancer.

[59] Gay-Bellile M., Véronèse L., Combes P., Eymard-Pierre E., Kwiatkowski F., Dauplat M.-M., Cayre A., Privat M., Abrial C., Bignon Y.-J. (2017). *TERT* promoter status and gene copy number gains: Effect on *TERT* expression and association with prognosis in breast cancer. Oncotarget.

[60] Zhang X., Klamer B., Li J., Fernandez S., Li L. (2020). A pan-cancer study of class-3 semaphorins as therapeutic targets in cancer. BMC Med. Genom..

