# Supplementary material for: Identification of Altered Primary Immunodeficiency-Associated Genes and Their Implications in Pediatric Cancers
Source: Cancers (Basel). 2022 Nov 30;14(23):5942. doi: 10.3390/cancers14235942 (PMC9741011; doi:10.3390/cancers14235942)
Supplement: Supplementary file 1 [file cancers-14-05942-s001.zip › Supplementary Information.docx]

**Identification of Altered Primary Immunodeficiency-Associated Genes and Their Implications in Pediatric Cancers**

**Appendix A-K**

**Supplementary Figures and Tables**

**APPENDIX A: Differential expression analysis (DEA) results using the *limma-voom* pipeline**


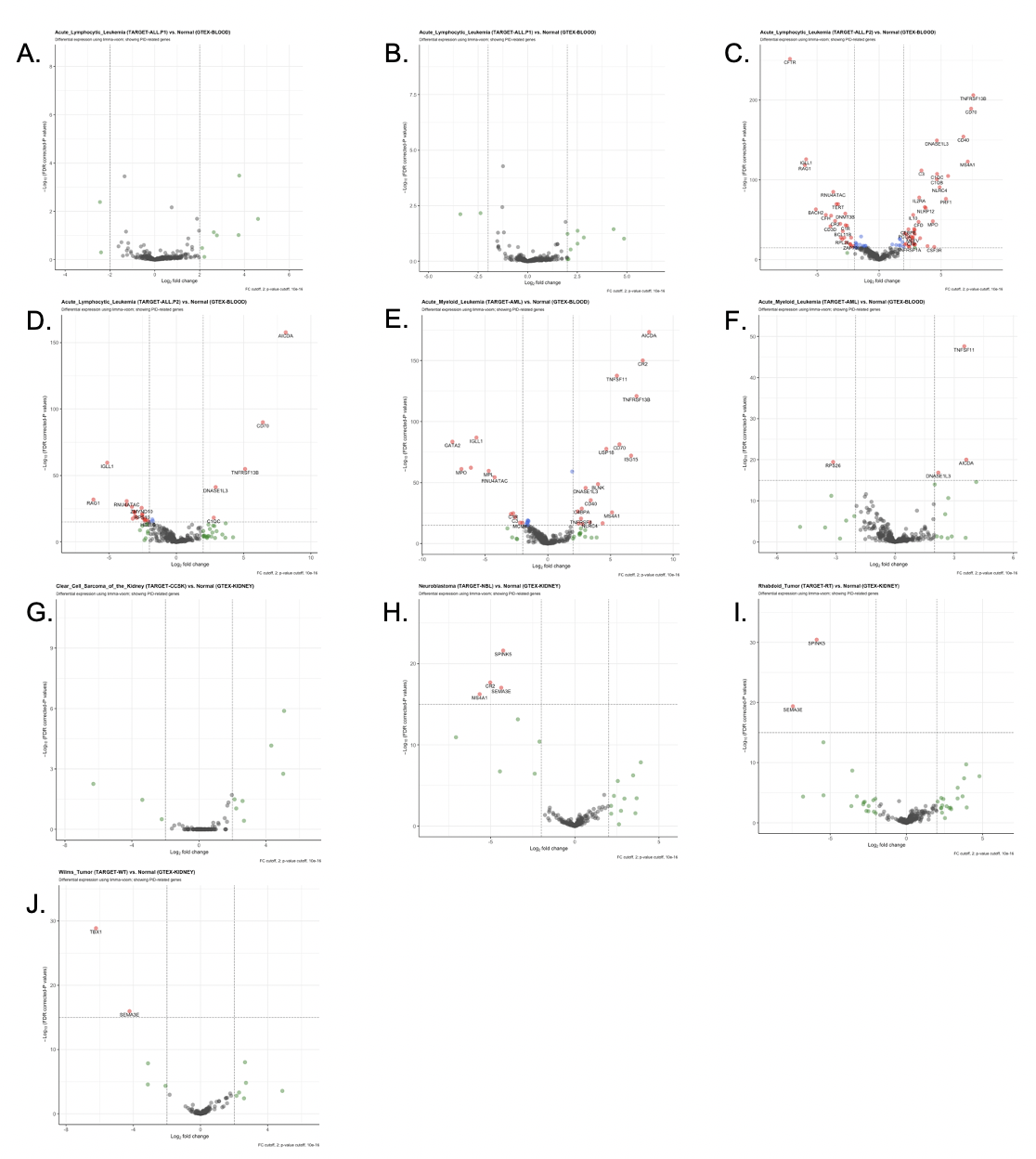


**Figure S1:** *EnhancedVolcano* plots produced from the DEA of PID-related genes for the A) ALL-P1 (TBM), B) ALL-P1 (TB), C) ALL-P2 (TBM), D) ALL-P2 (TB), E) AML (TBM), F) AML (TB), G) CCSK, H) NBL, I) RT, and J) WT TARGET tumor samples against the GTEx-blood or kidney normal tissue samples depending on cancer type. This analysis was performed with the *TCGAbiolinks* R package using the *limma-voom* pipeline.

**APPENDIX B: Differential expression analysis (DEA) using the *edgeR* pipeline**


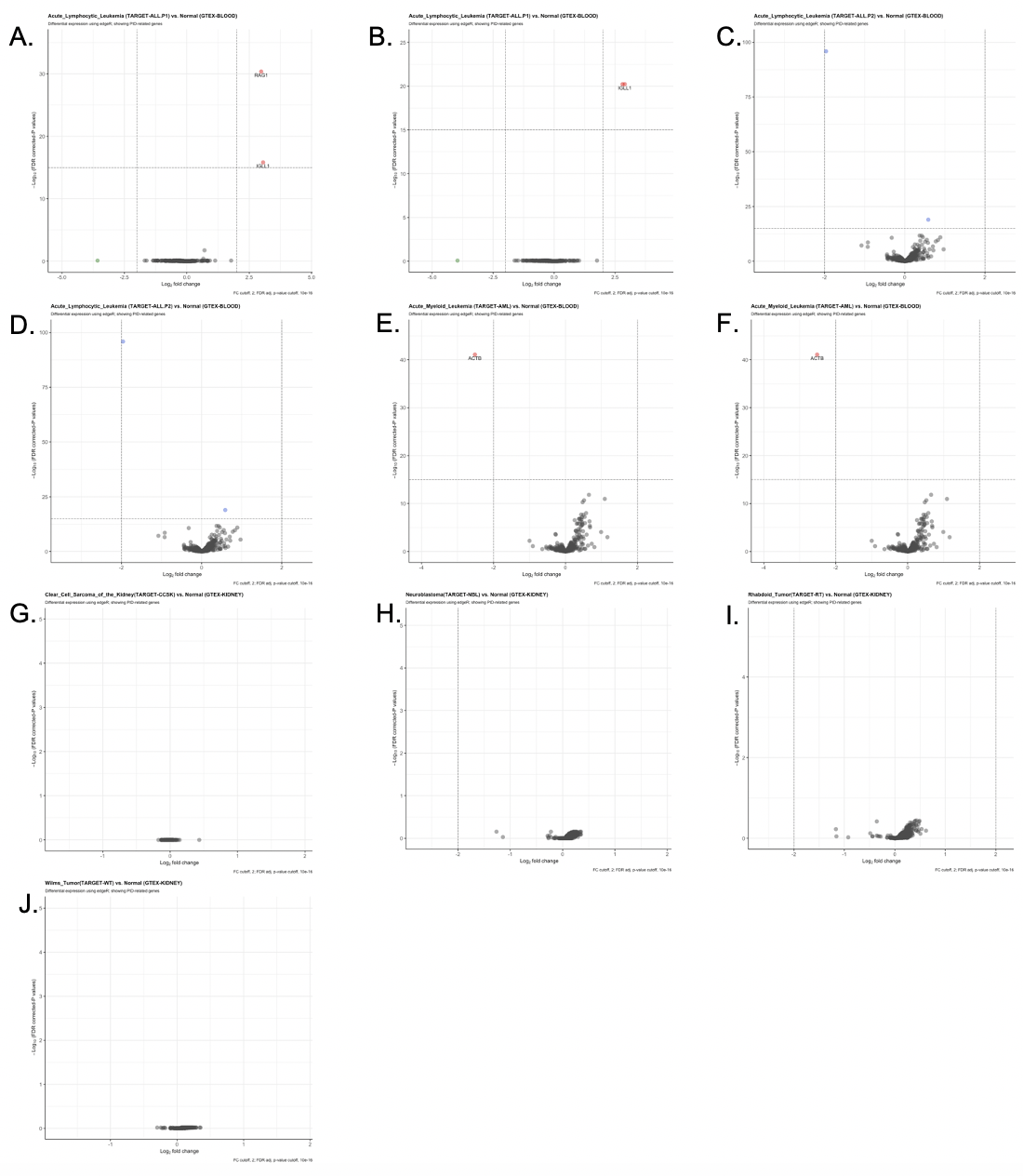


**Figure S2:** *EnhancedVolcano* plots produced from the DEA of PID genes for the A) ALL-P1 (TBM), B) ALL-P1 (TB), C) ALL-P2 (TBM), D) ALL-P2 (TB), E) AML (TBM), F) AML (TB), G) CCSK, H) NBL, I) RT, and J) WT TARGET tumor samples against the GTEx-blood or kidney normal tissue tissues depending on cancer type. This analysis was performed with the *TCGAbiolinks* R package using the *edgeR* pipeline.

**APPENDIX C: Summarized differential expression analysis (DEA) using the *limma-voom* pipeline for all the differentially expressed genes.**

**
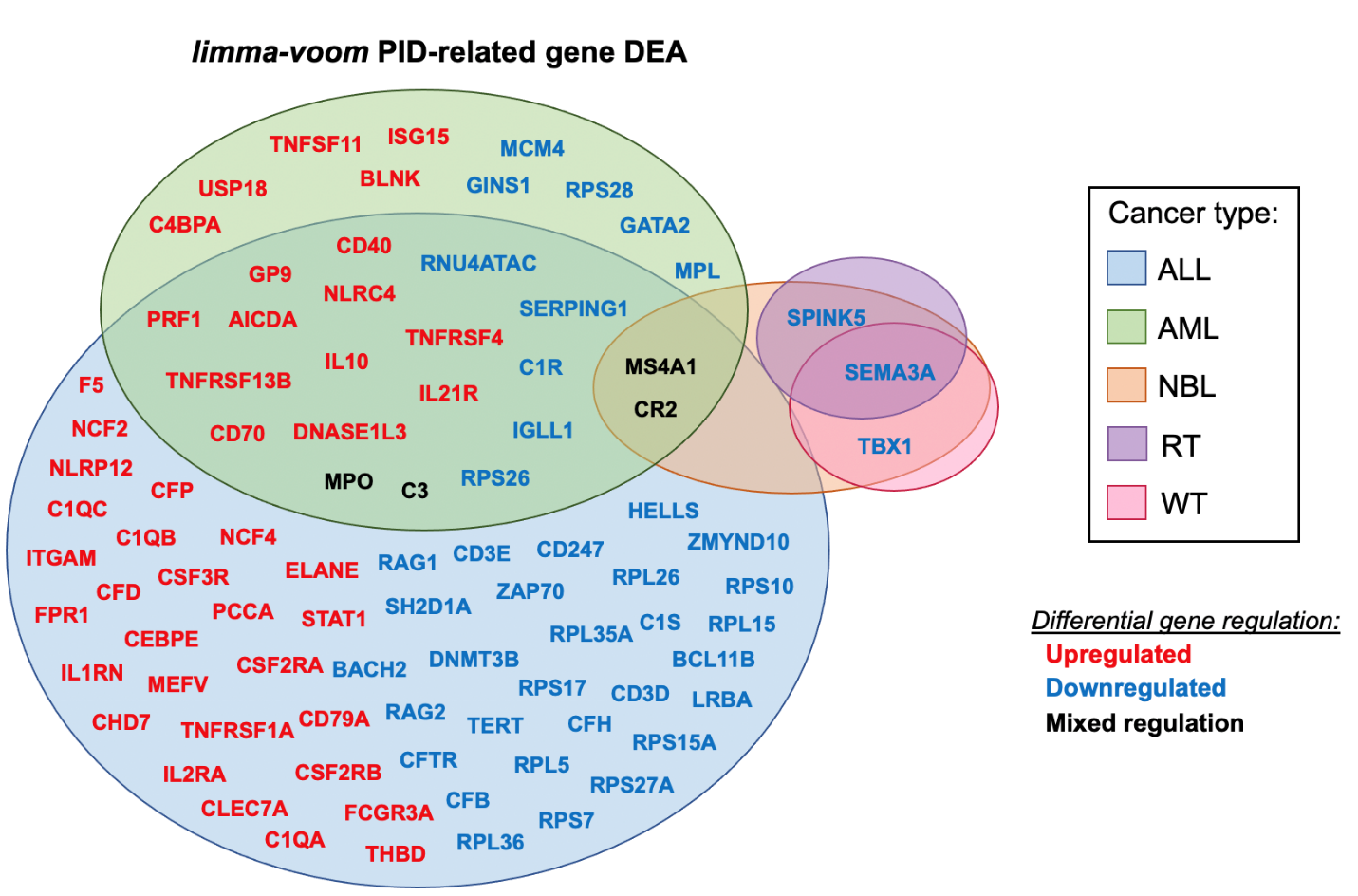
**

**Figure S3:** Venn diagram summary of DEA results for PID-related genes that are significantly differentially expressed in pediatric cancer types including AML, ALL, WT, RT, and NBL. The different gene names are listed in each of the cancer types in which they were significantly differentially expressed in. The colour of the gene represents the differential expression of each gene either upregulated (red), downregulated (blue), or mixed (black) if the gene had mixed regulation in different cancer types. The analyses have been performed using the *TCGAbiolinks* R package and the *limma-voom* pipeline.

**APPENDIX D: Summarized DEA results using the *limma-voom* and *edgeR* pipelines for all the differentially expressed genes and cancer types**

**Table S1:** Summary of DEA results for PID-related genes that are significantly differentially expressed in pediatric cancer types including AML, ALL, WT, RT, and NBL. Analyses were performed using the *TCGAbiolinks* R package and the *limma-voom* (use adj. *p*-value) or *edgeR* (use FDR) pipeline.

| **Gene Name** | **Cancer Type** | **Regulation** | **logFC** | **adj. p-value** | **Pipeline** |
| --- | --- | --- | --- | --- | --- |
| SEMA3A | WT | down | -4.2218 | 1.12E-16 | limma-voom |
|  | RT | down | -7.4468 | 4.15E-20 | limma-voom |
|  | NBL | down | -4.3708 | 9.14E-18 | limma-voom |
| MS4A1 | NBL | down | -5.6520 | 5.78E-17 | limma-voom |
|  | AML (TBM) | up | 5.1189 | 2.67E-26 | limma-voom |
|  | ALL (TB) | down | -3.2401 | 2.95E-18 | limma-voom |
|  | ALL (TBM) | up | 7.1488 | 1.29E-123 | limma-voom |
| CR2 | NBL | down | -5.0277 | 2.12E-18 | limma-voom |
|  | AML (TBM) | up | 7.5685 | 7.40E-151 | limma-voom |
|  | ALL (TBM) | down | -3.5445 | 2.36E-49 | limma-voom |
| SPINK5 | RT | down | -5.8893 | 3.52E-31 | limma-voom |
|  | NBL | down | -5.0277 | 2.42E-22 | limma-voom |
| AICDA | AML (TBM) | up | 8.0768 | 3.78E-174 | limma-voom |
|  | AML (TB) | up | 3.6138 | 9.27E-21 | limma-voom |
|  | ALL (TB) | up | 8.1305 | 2.18E-158 | limma-voom |
| TNFRSF13B | AML (TBM) | up | 7.0709 | 1.37E-121 | limma-voom |
|  | ALL (TBM) | up | 7.5940 | 6.95E-207 | limma-voom |
|  | ALL (TB) | up | 5.1080 | 1.52E-55 | limma-voom |
| CD70 | AML (TBM) | up | 5.7140 | 3.87E-82 | limma-voom |
|  | AML (TB) | up | 4.1147 | 2.55E-15 | limma-voom |
|  | ALL (TBM) | up | 7.4212 | 4.51E-190 | limma-voom |
|  | ALL (TB) | up | 6.4286 | 8.79E-91 | limma-voom |
| DNASE1L3 | AML (TBM) | up | 3.0153 | 2.57E-46 | limma-voom |
|  | AML (TB) | up | 2.1936 | 1.33E-17 | limma-voom |
|  | ALL (TBM) | up | 4.6634 | 3.54E-150 | limma-voom |
|  | ALL (TB) | up | 2.9224 | 5.49E-42 | limma-voom |
| CD40 | AML (TBM) | up | 3.4236 | 3.10E-36 | limma-voom |
|  | ALL (TBM) | up | 6.7991 | 5.75E-155 | limma-voom |
| NLRC4 | AML (TBM) | up | 3.3452 | 3.57E-18 | limma-voom |
|  | ALL (TBM) | up | 4.8854 | 1.78E-91 | limma-voom |
| C1R | AML (TBM) | down | -2.7625 | 1.76E-25 | limma-voom |
|  | ALL (TBM) | down | -2.7057 | 7.75E-44 | limma-voom |
| C3 | AML (TBM) | down | -2.6169 | 2.28E-22 | limma-voom |
|  | ALL (TBM) | up | 3.4192 | 1.67E-112 | limma-voom |
| MPO | AML (TBM) | down | -6.8905 | 7.88E-62 | limma-voom |
|  | ALL (TBM) | up | 4.3337 | 7.03E-49 | limma-voom |
| IGLL1 | AML (TBM) | down | -5.6949 | 1.32E-87 | limma-voom |
|  | ALL (TBM) | down | -5.8822 | 1.40E-126 | limma-voom |
|  | ALL (TB) | down | -5.1328 | 2.60E-60 | limma-voom |
|  | ALL (TBM) | up | 3.0554 | 1.52E-16 | edgeR |
|  | ALL (TB) | up | 2.8997 | 5.92E-21 | edgeR |
| RNU4ATAC | AML (TBM) | down | -4.2409 | 4.84E-55 | limma-voom |
|  | ALL (TBM) | down | -3.6982 | 9.22E-86 | limma-voom |
|  | ALL (TB) | down | -3.6840 | 2.13E-31 | limma-voom |
| TNFRSF4 | AML (TBM) | up | 2.6427 | 3.60E-21 | limma-voom |
|  | ALL (TBM) | up | 2.8291 | 3.03E-39 | limma-voom |
| IL21R | AML (TBM) | up | 2.4488 | 3.33E-17 | limma-voom |
|  | ALL (TBM) | up | 2.8781 | 9.60E-33 | limma-voom |
| IL10 | AML (TBM) | up | 2.3891 | 5.01E-27 | limma-voom |
|  | ALL (TBM) | up | 2.7437 | 8.21E-57 | limma-voom |
| SERPING1 | AML (TBM) | down | -2.9394 | 6.54E-25 | limma-voom |
|  | ALL (TBM) | down | -3.1329 | 9.72E-46 | limma-voom |
| GP9 | AML (TBM) | up | 2.7555 | 1.02E-16 | limma-voom |
|  | ALL (TBM) | up | 2.2211 | 2.78E-32 | limma-voom |
| PRF1 | AML (TBM) | up | 4.3686 | 2.43E-17 | limma-voom |
|  | ALL (TBM) | up | 5.3925 | 5.69E-77 | limma-voom |
| RPS26 | AML (TBM) | down | -6.1334 | 7.09E-63 | limma-voom |
|  | AML (TB) | down | -3.1422 | 3.61E-20 | limma-voom |
|  | ALL (TBM) | down | -3.8693 | 8.25E-56 | limma-voom |
| TBX1 | WT | down | -6.2210 | 1.37E-29 | limma-voom |
|  | NBL | down | -3.3868 | 7.26E-14 | limma-voom |
| TNFSF11 | AML (TBM) | up | 5.5067 | 2.32E-138 | limma-voom |
|  | AML (TB) | up | 3.5088 | 2.48E-48 | limma-voom |
| USP18 | AML (TBM) | up | 4.6657 | 2.66E-78 | limma-voom |
| ISG15 | AML (TBM) | up | 6.6439 | 8.48E-73 | limma-voom |
| BLNK | AML (TBM) | up | 4.0029 | 1.93E-49 | limma-voom |
| C4BPA | AML (TBM) | up | 2.7065 | 2.29E-29 | limma-voom |
| MCM4 | AML (TBM) | down | -2.2166 | 8.97E-18 | limma-voom |
| MPL | AML (TBM) | down | -4.7167 | 2.79E-60 | limma-voom |
| GATA2 | AML (TBM) | down | -7.6078 | 3.28E-84 | limma-voom |
| RPS28 | AML (TBM) | down | -2.0278 | 9.03E-18 | limma-voom |
| GINS1 | AML (TB) | down | 2.0131 | 1.04E-14 | limma-voom |
| ACTB | AML (TBM) | down | -2.5160 | 8.32E-42 | edgeR |
|  | AML (TB) | down | -2.5160 | 8.32E-42 | edgeR |
| C1QC | ALL (TBM) | up | 4.6805 | 3.76E-108 | limma-voom |
|  | ALL (TB) | up | 2.7775 | 6.29E-19 | limma-voom |
| C1QB | ALL (TBM) | up | 4.6619 | 1.28E-101 | limma-voom |
| CFD | ALL (TBM) | up | 3.1881 | 4.45E-48 | limma-voom |
| CSF3R | ALL (TBM) | up | 4.4505 | 1.20E-16 | limma-voom |
| CEBPE | ALL (TBM) | up | 2.3709 | 9.39E-39 | limma-voom |
| PCCA | ALL (TBM) | up | 2.0636 | 1.22E-33 | limma-voom |
| MEFV | ALL (TBM) | up | 2.7155 | 8.56E-29 | limma-voom |
| CFP | ALL (TBM) | up | 2.6215 | 1.85E-24 | limma-voom |
| TNFRSF1A | ALL (TBM) | up | 2.4331 | 6.68E-17 | limma-voom |
| ZAP70 | ALL (TBM) | down | -2.3187 | 9.03E-20 | limma-voom |
| RPL26 | ALL (TBM) | down | -2.9691 | 2.92E-26 | limma-voom |
|  | ALL (TB) | down | -3.0647 | 3.04E-20 | limma-voom |
| BCL11B | ALL (TBM) | down | -2.9304 | 2.19E-36 | limma-voom |
| CD3D | ALL (TBM) | down | -3.9298 | 1.35E-42 | limma-voom |
| CFH | ALL (TBM) | down | -4.2923 | 9.51E-57 | limma-voom |
| DNMT3B | ALL (TBM) | down | -2.7250 | 1.53E-58 | limma-voom |
| BACH2 | ALL (TBM) | down | -5.0969 | 9.54E-64 | limma-voom |
| TERT | ALL (TBM) | down | -3.3039 | 2.13E-70 | limma-voom |
| RAG1 | ALL (TBM) | down | -5.9868 | 4.14E-119 | limma-voom |
|  | ALL (TB) | down | -6.1586 | 1.26E-32 | limma-voom |
|  | ALL (TBM) | up | 2.9818 | 4.37E-31 | edgeR |
|  | ALL (TB) | up | 2.8082 | 5.92E-21 | edgeR |
| CFTR | ALL (TBM) | down | -7.1867 | 2.10E-252 | limma-voom |
| NCF4 | ALL (TBM) | up | 2.8218 | 2.46E-19 | limma-voom |
| CSF2RB | ALL (TBM) | up | 2.0618 | 2.11E-19 | limma-voom |
| CD79A | ALL (TBM) | up | 3.3076 | 1.49E-27 | limma-voom |
| STAT1 | ALL (TBM) | up | 2.1834 | 1.01E-20 | limma-voom |
| NCF2 | ALL (TBM) | up | 2.8892 | 1.07E-15 | limma-voom |
| IL2RA | ALL (TBM) | up | 3.2353 | 1.15E-78 | limma-voom |
| IL1RN | ALL (TBM) | up | 2.8734 | 5.13E-38 | limma-voom |
| NLRP12 | ALL (TBM) | up | 3.7601 | 1.47E-65 | limma-voom |
| ITGAM | ALL (TBM) | up | 2.7566 | 9.59E-21 | limma-voom |
| FPR1 | ALL (TBM) | up | 3.9077 | 9.03E-18 | limma-voom |
| CHD7 | ALL (TBM) | up | 2.3687 | 6.50E-18 | limma-voom |
| RPS7 | ALL (TBM) | down | -2.3373 | 3.32E-20 | limma-voom |
|  | ALL (TB) | down | -3.1086 | 6.19E-23 | limma-voom |
| CLEC7A | ALL (TBM) | up | 2.6970 | 6.64E-28 | limma-voom |
| C1QA | ALL (TBM) | up | 3.6911 | 1.09E-66 | limma-voom |
| RAG2 | ALL (TBM) | down | -3.4413 | 2.08E-70 | limma-voom |
|  | ALL (TB) | down | -3.7064 | 1.51E-28 | limma-voom |
| THBD | ALL (TBM) | up | 2.3258 | 4.01E-28 | limma-voom |
| C1S | ALL (TBM) | down | -2.5855 | 2.82E-43 | limma-voom |
| RPS17 | ALL (TBM) | down | -2.2140 | 4.47E-17 | limma-voom |
|  | ALL (TB) | down | -2.9461 | 7.80E-19 | limma-voom |
| RPL35A | ALL (TBM) | down | -2.0418 | 6.47E-18 | limma-voom |
|  | ALL (TB) | down | -3.1233 | 1.96E-23 | limma-voom |
| SH2D1A | ALL (TBM) | down | -3.0901 | 2.95E-28 | limma-voom |
| ELANE | ALL (TBM) | up | 5.5616 | 1.25E-105 | limma-voom |
|  | ALL (TB) | up | 3.6678 | 1.07E-14 | limma-voom |
| CSF2RA | ALL (TBM) | up | 2.5606 | 9.44E-30 | limma-voom |
| F5 | ALL (TBM) | up | 2.8027 | 3.55E-35 | limma-voom |
| CD247 | ALL (TBM) | down | -2.6360 | 3.23E-22 | limma-voom |
| CD3E | ALL (TBM) | down | -2.8075 | 3.15E-28 | limma-voom |
| FCGR3A | ALL (TBM) | up | 2.9076 | 9.34E-20 | limma-voom |
| CFB | ALL (TBM) | down | -2.2498 | 2.85E-28 | limma-voom |
| HELLS | ALL (TB) | down | -2.0986 | 2.47E-16 | limma-voom |
| ZMYND10 | ALL (TB) | down | -2.5784 | 2.64E-26 | limma-voom |
| RPS10 | ALL (TB) | down | -2.5350 | 9.40E-22 | limma-voom |
| RPL15 | ALL (TB) | down | -2.3524 | 6.22E-18 | limma-voom |
| LRBA | ALL (TB) | down | -3.2988 | 3.57E-27 | limma-voom |
| RPL36 | ALL (TB) | down | -2.3509 | 1.12E-16 | limma-voom |
| RPS15A | ALL (TB) | down | -2.4936 | 1.05E-18 | limma-voom |
| RPS27A | ALL (TB) | down | -2.5291 | 8.07E-21 | limma-voom |
| RPL5 | ALL (TB) | down | -2.2563 | 2.10E-17 | limma-voom |

**APPENDIX E: GO enrichment analysis results using the *topGO* package**

**Table S2:** GO ID and terms from the biological process GO enrichment analysis of all differentially expressed genes TARGET tumor samples against the GTEx normal tissue samples. This analysis was performed with the *topGO* R package.

| **Cancer tissue** | **GO ID** | **Term description** | **KS** |
| --- | --- | --- | --- |
| ALL (TB) | GO:0034612 | response to tumor necrosis factor | 0.0007 |
|  | GO:0071356 | cellular response to tumor necrosis factor | 0.0007 |
|  | GO:0019221 | cytokine-mediated signaling pathway | 0.0010 |
|  | GO:0098655 | cation transmembrane transport | 0.0027 |
|  | GO:0071345 | cellular response to cytokine stimulus | 0.0028 |
|  | GO:0034097 | response to cytokine | 0.0042 |
|  | GO:0042221 | response to chemical | 0.0047 |
|  | GO:0006812 | cation transport | 0.0066 |
|  | GO:0006816 | calcium ion transport | 0.0068 |
|  | GO:0034220 | ion transmembrane transport | 0.0099 |
| ALL (TBM) | GO:0022618 | ribonucleoprotein complex assembly | 0.0001 |
|  | GO:0071826 | ribonucleoprotein complex subunit organization | 0.0001 |
|  | GO:0006397 | mRNA processing | 0.0003 |
|  | GO:0000244 | spliceosomal tri-snRNP complex assembly | 0.0017 |
|  | GO:0000387 | spliceosomal snRNP assembly | 0.0017 |
| AML (TB) | GO:0006811 | ion transport | 0.0450 |
| AML (TBM) | GO:0090304 | nucleic acid metabolic process | 0.0002 |
|  | GO:0016070 | RNA metabolic process | 0.0004 |
|  | GO:0006357 | regulation of transcription by RNA polymerase II | 0.0004 |
|  | GO:0006366 | transcription by RNA polymerase II | 0.0004 |
|  | GO:0009790 | embryo development | 0.0005 |
|  | GO:0001501 | skeletal system development | 0.0008 |
|  | GO:0048704 | embryonic skeletal system morphogenesis | 0.0008 |
|  | GO:0048562 | embryonic organ morphogenesis | 0.0008 |
|  | GO:0048568 | embryonic organ development | 0.0009 |
|  | GO:0048706 | embryonic skeletal system development | 0.0010 |
| NBL | GO:0008202 | steroid metabolic process | 0.0001 |
|  | GO:0044281 | small molecule metabolic process | 0.0002 |
|  | GO:0008610 | lipid biosynthetic process | 0.0003 |
|  | GO:0006720 | isoprenoid metabolic process | 0.0007 |
|  | GO:0006721 | terpenoid metabolic process | 0.0007 |
|  | GO:1901615 | organic hydroxy compound metabolic process | 0.0007 |
|  | GO:0043687 | post-translational protein modification | 0.0010 |
|  | GO:0044255 | cellular lipid metabolic process | 0.0013 |
|  | GO:0016192 | vesicle-mediated transport | 0.0015 |
| RT | GO:0006952 | defense response | 0.0028 |
|  | GO:0031424 | keratinization | 0.0053 |
|  | GO:0070268 | cornification | 0.0053 |
|  | GO:0015850 | organic hydroxy compound transport | 0.0068 |
|  | GO:0030198 | extracellular matrix organization | 0.0078 |
|  | GO:0043062 | extracellular structure organization | 0.0078 |
|  | GO:0060627 | regulation of vesicle-mediated transport | 0.0078 |
|  | GO:0006508 | proteolysis | 0.0090 |
|  | GO:0043170 | macromolecule metabolic process | 0.0096 |
|  | GO:0010469 | regulation of signaling receptor activity | 0.0139 |
| WT | GO:0032501 | multicellular organismal process | 0.0001 |
|  | GO:0003008 | system process | 0.0002 |
|  | GO:0030029 | actin filament-based process | 0.0002 |
|  | GO:0048468 | cell development | 0.0006 |
|  | GO:0032989 | cellular component morphogenesis | 0.0007 |
|  | GO:0030049 | muscle filament sliding | 0.0007 |
|  | GO:0033275 | actin-myosin filament sliding | 0.0007 |
|  | GO:0055002 | striated muscle cell development | 0.0010 |

**APPENDIX F: GO enrichment analysis results using the *topGO* package for PID-related genes**

**Table S3:** GO ID and terms from the biological process GO enrichment analysis of all PID-related differentially expressed genes TARGET tumor samples against the GTEx normal tissue samples. This analysis was performed with the *topGO* R package.

| **Cancer tissue** | **GO ID** | **Term description** |
| --- | --- | --- |
| ALL (TB) | GO:0001775 | cell activation |
|  | GO:0045321 | leukocyte activation |
| ALL (TBM) | GO:0016043 | cellular component organization |
|  | GO:0019724 | B cell mediated immunity |
|  | GO:0002449 | lymphocyte mediated immunity |
|  | GO:0016064 | immunoglobulin mediated immune response |
|  | GO:0002443 | leukocyte mediated immunity |
|  | GO:0071840 | cellular component organization or biogenesis |
|  | GO:0002250 | adaptive immune response |
|  | GO:0006959 | humoral immune response |
|  | GO:0006956 | complement activation |
| AML (TBM) | GO:0002520 | immune system development |
|  | GO:0002682 | regulation of immune system process |
|  | GO:0010033 | response to organic substance |
|  | GO:0042221 | response to chemical |
|  | GO:0030097 | hemopoiesis |
|  | GO:0048513 | animal organ development |
|  | GO:0048534 | hematopoietic or lymphoid organ development |
|  | GO:0032502 | developmental process |
|  | GO:0048856 | anatomical structure development |
|  | GO:0051716 | cellular response to stimulus |

**APPENDIX G: Copy number variant analysis of all PID-related genes using cBioPortal**

**Table S4:** Summary copy number variant analysis for 472 PID-related genes in all the following cancer types: ALL, AML, NBL and WT. Attached file: Supplementary_Data_1.xlsx.

**APPENDIX H: Survival analysis of all, and mutated PID-related genes using cBioPortal**


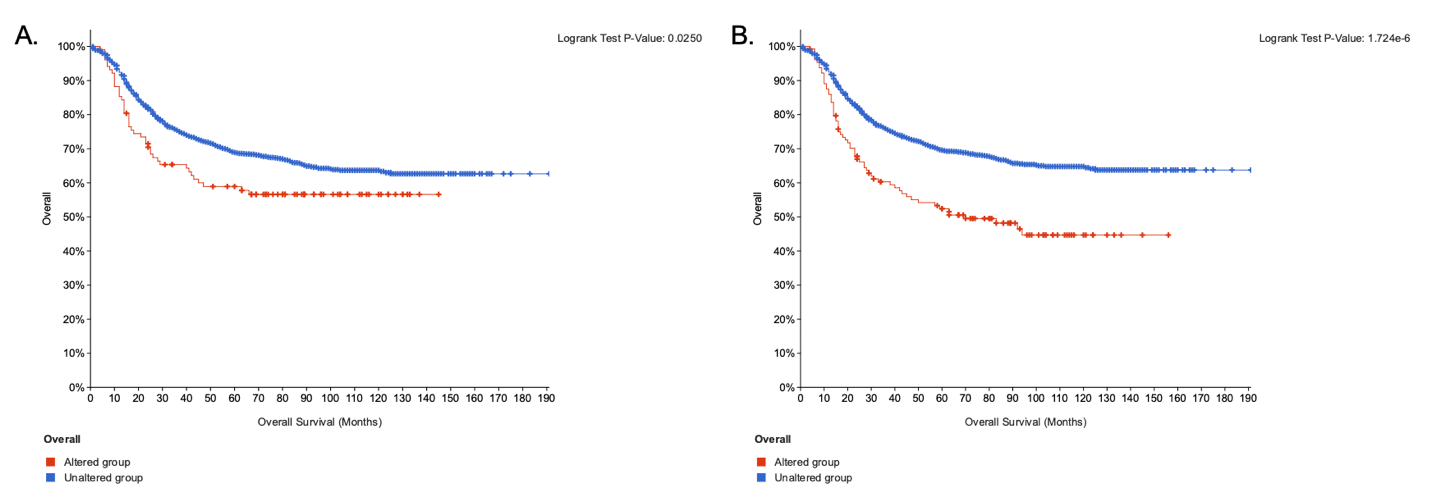


**Figure S4:** Kaplan-Meier survival curve summary for A) 472 PID-related genes (*p*=0.025) and B) 20 genes mutated in >0.1% of patients (*p*=1.724e-6). The altered group is defined as harboring somatic mutations from whole genome or whole exome sequencing. Only patients with mutations data were used from the portal in this analysis. *P*<0.05 was considered to indicate statistical significance. Analysis was done using cBioPortal including the following TARGET projects: ALL-P2, AML, NBL, RT, and WT.

**APPENDIX I: Survival analysis summary of all differentially expressed genes and pediatric cancer types**

**Table S5:** Summary survival analysis results for 89 differentially expressed genes in the following cancer types: ALL, AML, CCSK, NBL, RT, and WT. This analysis was done using Cox regression analysis with a generated R script. Attached file: Supplementary_Data_2.xlsx.

**
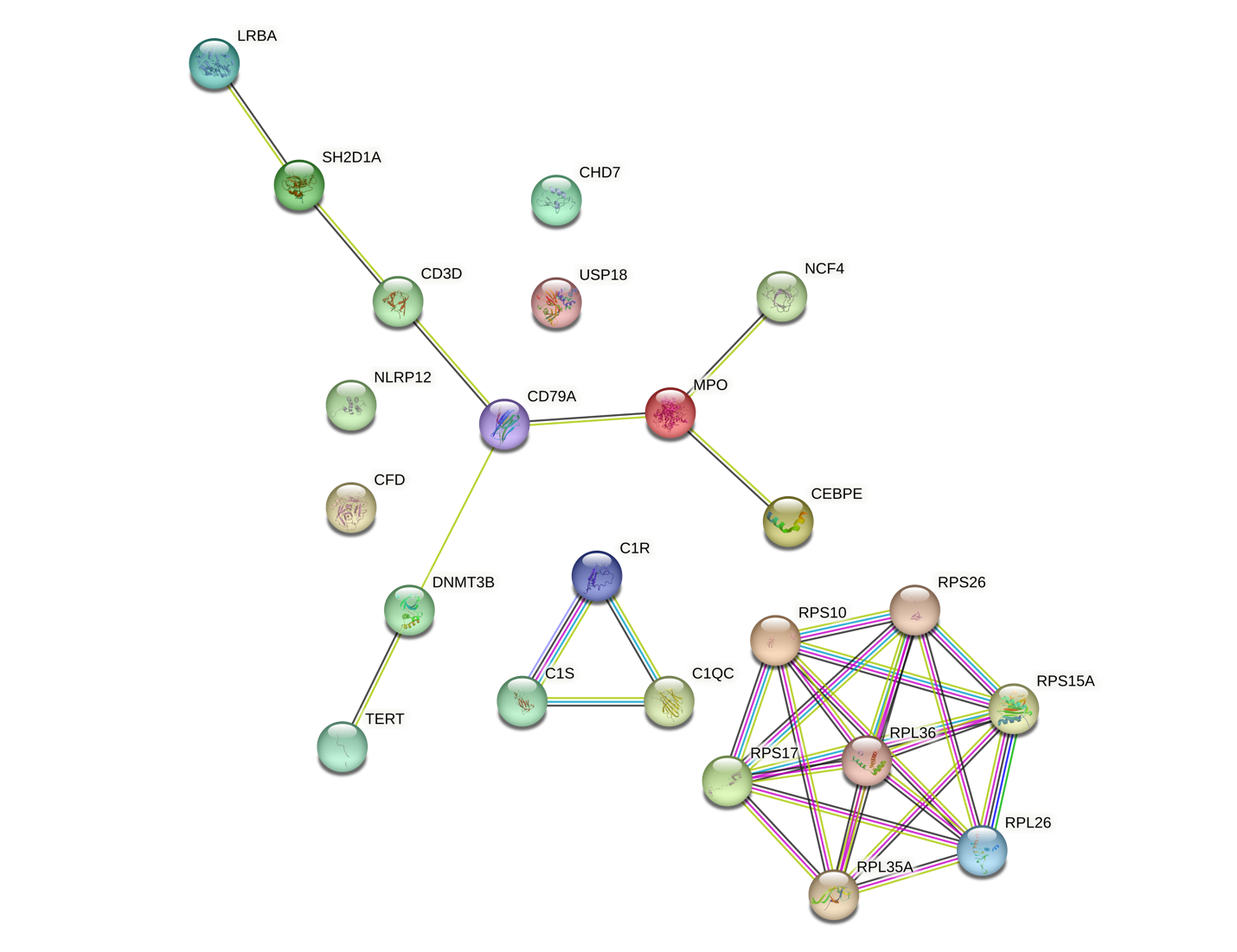
**

**Figure S5:** STRING network visualization retrieved for the 23 PID-related genes with significant effects on overall survival in at least two different pediatric cancer types. STRING (v11) online platform was used for analysis.

**Table S6:** Summary results from the biological process GO enrichment analysis of the 23 PID-related genes with significant effects on overall survival in at least two different pediatric cancer types. This analysis was performed using STRING (v11).

| **GO ID** | **Term description** | **Strength** | **FDR** |
| --- | --- | --- | --- |
| GO:0000184 | Nuclear-transcribed mrna catabolic process, nonsense-mediated decay | 1.7 | 2.66E-07 |
| GO:0006614 | SRP-dependent cotranslational protein targeting to membrane | 1.79 | 2.66E-07 |
| GO:0019083 | Viral transcription | 1.71 | 2.66E-07 |
| GO:0044419 | Interspecies interaction between organisms | 0.83 | 2.82E-07 |
| GO:0006413 | Translational initiation | 1.63 | 3.67E-07 |
| GO:0072594 | Establishment of protein localization to organelle | 1.2 | 1.81E-05 |
| GO:0006956 | Complement activation | 1.8 | 0.00031 |
| GO:0002376 | Immune system process | 0.65 | 0.00038 |
| GO:0002250 | Adaptive immune response | 1.21 | 0.00064 |
| GO:0002181 | Cytoplasmic translation | 1.67 | 0.0007 |
| GO:0002253 | Activation of immune response | 1.12 | 0.0018 |
| GO:0050778 | Positive regulation of immune response | 0.99 | 0.0018 |
| GO:0050776 | Regulation of immune response | 0.88 | 0.0019 |
| GO:0044265 | Cellular macromolecule catabolic process | 0.87 | 0.0022 |
| GO:0010467 | Gene expression | 0.66 | 0.0024 |
| GO:0051707 | Response to other organism | 0.79 | 0.0024 |
| GO:0006958 | Complement activation, classical pathway | 1.83 | 0.0045 |
| GO:0006959 | Humoral immune response | 1.19 | 0.0047 |
| GO:0042254 | Ribosome biogenesis | 1.16 | 0.0059 |
| GO:0010629 | Negative regulation of gene expression | 0.63 | 0.0114 |
| GO:0016070 | RNA metabolic process | 0.68 | 0.0114 |
| GO:0030449 | Regulation of complement activation | 1.66 | 0.0114 |
| GO:0009059 | Macromolecule biosynthetic process | 0.67 | 0.0138 |
| GO:0098542 | Defense response to other organism | 0.82 | 0.0138 |
| GO:0050789 | Regulation of biological process | 0.21 | 0.0186 |
| GO:0090304 | Nucleic acid metabolic process | 0.59 | 0.0193 |
| GO:0044248 | Cellular catabolic process | 0.64 | 0.0218 |
| GO:0006364 | rRNA processing | 1.21 | 0.0231 |
| GO:0048584 | Positive regulation of response to stimulus | 0.58 | 0.024 |
| GO:0009605 | Response to external stimulus | 0.57 | 0.0275 |
| GO:0044271 | Cellular nitrogen compound biosynthetic process | 0.65 | 0.0428 |
| GO:0045730 | Respiratory burst | 1.98 | 0.0454 |

**APPENDIX J: Pathway analysis for the 89 PID-related differentially expressed genes**


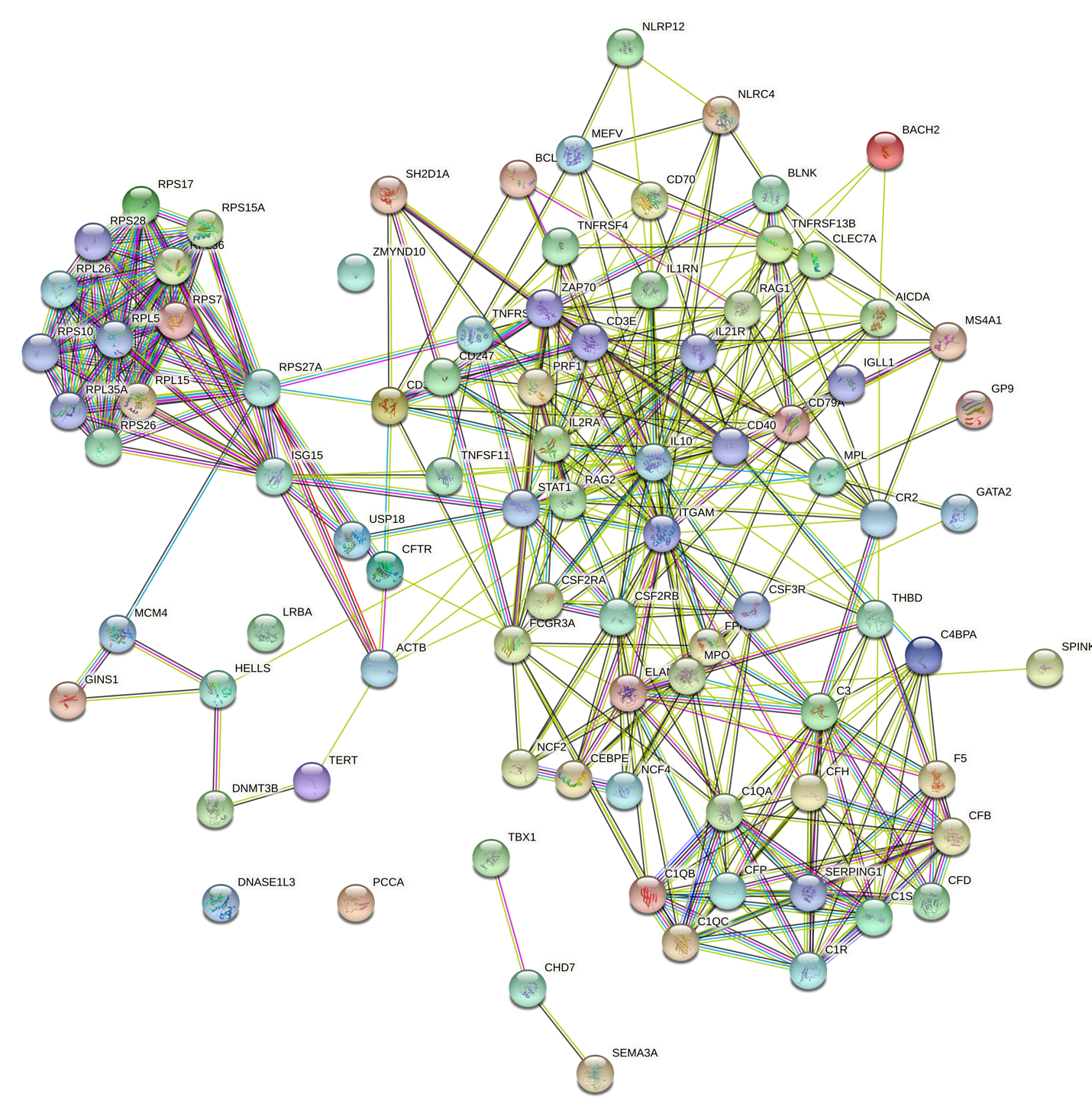


**Figure S6:** STRING network visualization retrieved for the 89 PID-related differentially expressed genes in pediatric cancers. STRING (v11) online platform was used for analysis.

**Table S7:** Summary results from the biological process GO enrichment analysis of the 89 PID-related differentially expressed genes. Only the top 40 terms are included in this table. This analysis was performed using STRING (v11).

| **GO ID** | **Term description** | **Strength** | **FDR** |
| --- | --- | --- | --- |
| GO:0002376 | immune system process | 0.76 | 6.25E-32 |
| GO:0006955 | immune response | 0.83 | 4.15E-25 |
| GO:0002684 | positive regulation of immune system process | 0.98 | 1.52E-24 |
| GO:0002682 | regulation of immune system process | 0.84 | 4.71E-23 |
| GO:0050776 | regulation of immune response | 0.95 | 2.02E-21 |
| GO:0050778 | positive regulation of immune response | 1.05 | 1.03E-20 |
| GO:0002253 | activation of immune response | 1.17 | 2.63E-20 |
| GO:0006952 | defense response | 0.84 | 6.24E-20 |
| GO:0051707 | response to other organism | 0.85 | 1.12E-19 |
| GO:0098542 | defense response to other organism | 0.92 | 1.11E-18 |
| GO:0006956 | complement activation | 1.8 | 2.75E-18 |
| GO:0031347 | regulation of defense response | 0.98 | 2.75E-18 |
| GO:0072376 | protein activation cascade | 1.65 | 8.41E-18 |
| GO:0009605 | response to external stimulus | 0.66 | 2.58E-17 |
| GO:0050727 | regulation of inflammatory response | 1.16 | 4.91E-17 |
| GO:0002697 | regulation of immune effector process | 1.13 | 1.85E-16 |
| GO:0002250 | adaptive immune response | 1.2 | 4.11E-16 |
| GO:0032101 | regulation of response to external stimulus | 0.84 | 1.53E-15 |
| GO:0048584 | positive regulation of response to stimulus | 0.65 | 1.61E-15 |
| GO:0002673 | regulation of acute inflammatory response | 1.53 | 3.95E-15 |
| GO:0002920 | regulation of humoral immune response | 1.62 | 5.33E-15 |
| GO:0030449 | regulation of complement activation | 1.71 | 8.63E-15 |
| GO:0080134 | regulation of response to stress | 0.75 | 8.63E-15 |
| GO:2000257 | regulation of protein activation cascade | 1.69 | 1.20E-14 |
| GO:0006959 | humoral immune response | 1.2 | 1.69E-14 |
| GO:0001775 | cell activation | 0.8 | 6.31E-14 |
| GO:0006950 | response to stress | 0.51 | 7.84E-14 |
| GO:0048583 | regulation of response to stimulus | 0.47 | 8.06E-14 |
| GO:0045321 | leukocyte activation | 0.83 | 1.62E-13 |
| GO:0045087 | innate immune response | 0.9 | 2.62E-13 |
| GO:0046649 | lymphocyte activation | 1.07 | 3.16E-13 |
| GO:0006958 | complement activation, classical pathway | 1.82 | 3.19E-13 |
| GO:0002252 | immune effector process | 0.81 | 3.40E-13 |
| GO:0050896 | response to stimulus | 0.3 | 5.56E-13 |
| GO:0051704 | multi-organism process | 0.56 | 9.73E-13 |
| GO:0070613 | regulation of protein processing | 1.4 | 1.19E-12 |
| GO:0048518 | positive regulation of biological process | 0.38 | 2.03E-12 |
| GO:0006614 | SRP-dependent cotranslational protein targeting to membrane | 1.46 | 2.28E-12 |
| GO:0016064 | immunoglobulin mediated immune response | 1.53 | 5.55E-12 |
| GO:0065007 | biological regulation | 0.2 | 1.01E-11 |

**APPENDIX K: Pathway analysis for the 20 genes mutated in two or more pediatric cancer patients**


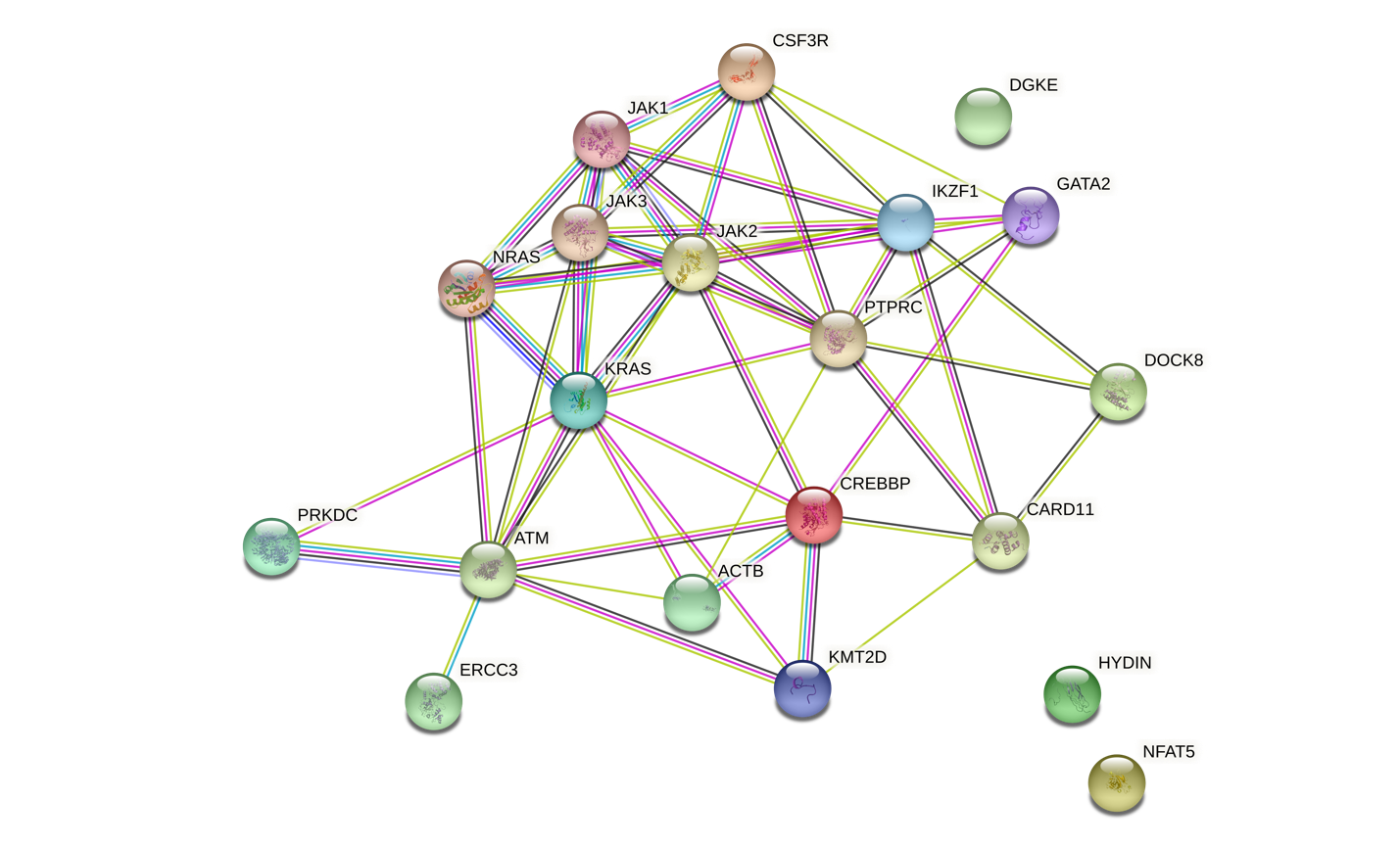


**Figure S7:** STRING network visualization retrieved for the 20 PID-related genes mutated in two or more pediatric cancer patients. STRING (v11) online platform was used for analysis.

**Table S8:** Summary results from the biological process GO enrichment analysis of the 20 PID-related genes mutated in two or more pediatric cancer patients. Only the top 40 terms are included in this table. This analysis was done using STRING (v11).

| **GO ID** | **Term description** | **Strength** | **FDR** |
| --- | --- | --- | --- |
| GO:0002682 | regulation of immune system process | 1.02 | 1.09E-10 |
| GO:0002376 | immune system process | 0.79 | 1.20E-07 |
| GO:0050776 | regulation of immune response | 1.09 | 1.20E-07 |
| GO:0002684 | positive regulation of immune system process | 1.05 | 1.71E-06 |
| GO:0002521 | leukocyte differentiation | 1.34 | 5.76E-06 |
| GO:0030097 | hemopoiesis | 1.17 | 6.99E-06 |
| GO:0048872 | homeostasis of number of cells | 1.48 | 7.69E-06 |
| GO:0045088 | regulation of innate immune response | 1.28 | 8.89E-06 |
| GO:0046649 | lymphocyte activation | 1.28 | 8.89E-06 |
| GO:0050778 | positive regulation of immune response | 1.12 | 9.03E-06 |
| GO:0002253 | activation of immune response | 1.24 | 9.71E-06 |
| GO:0002429 | immune response-activating cell surface receptor signaling pathway | 1.4 | 9.71E-06 |
| GO:0008284 | positive regulation of cell population proliferation | 1 | 9.71E-06 |
| GO:0016310 | phosphorylation | 0.9 | 9.71E-06 |
| GO:0030098 | lymphocyte differentiation | 1.41 | 9.71E-06 |
| GO:0042127 | regulation of cell population proliferation | 0.83 | 9.71E-06 |
| GO:0048518 | positive regulation of biological process | 0.48 | 1.34E-05 |
| GO:0002377 | immunoglobulin production | 1.92 | 1.35E-05 |
| GO:0031347 | regulation of defense response | 1.06 | 1.35E-05 |
| GO:0001775 | cell activation | 0.93 | 1.93E-05 |
| GO:0042113 | B cell activation | 1.53 | 2.14E-05 |
| GO:0050865 | regulation of cell activation | 1.13 | 2.76E-05 |
| GO:0050867 | positive regulation of cell activation | 1.28 | 2.95E-05 |
| GO:0002223 | stimulatory C-type lectin receptor signaling pathway | 1.8 | 2.99E-05 |
| GO:0042592 | homeostatic process | 0.82 | 3.25E-05 |
| GO:0042981 | regulation of apoptotic process | 0.81 | 3.34E-05 |
| GO:0035556 | intracellular signal transduction | 0.81 | 3.57E-05 |
| GO:0010604 | positive regulation of macromolecule metabolic process | 0.62 | 4.09E-05 |
| GO:0002218 | activation of innate immune response | 1.42 | 4.77E-05 |
| GO:0048584 | positive regulation of response to stimulus | 0.72 | 4.78E-05 |
| GO:0006796 | phosphate-containing compound metabolic process | 0.72 | 4.91E-05 |
| GO:0031349 | positive regulation of defense response | 1.21 | 5.26E-05 |
| GO:0045321 | leukocyte activation | 0.94 | 5.26E-05 |
| GO:0048583 | regulation of response to stimulus | 0.55 | 5.78E-05 |
| GO:0048870 | cell motility | 0.93 | 5.92E-05 |
| GO:0006468 | protein phosphorylation | 0.93 | 6.10E-05 |
| GO:0006950 | response to stress | 0.59 | 6.10E-05 |
| GO:0032101 | regulation of response to external stimulus | 0.91 | 7.40E-05 |
| GO:0007165 | signal transduction | 0.49 | 7.42E-05 |
| GO:0043900 | regulation of multi-organism process | 1.02 | 7.42E-05 |
